# Division of the large and multifunctional glycoside hydrolase family 2: high functional specificity and biochemical assays in the uncharacterized subfamilies

**DOI:** 10.1186/s13068-025-02669-8

**Published:** 2025-07-09

**Authors:** Annie Lebreton, Marie-Line Garron, Marlene Vuillemin, Bo Pilgaard, Bastian V. H. Hornung, Elodie Drula, Vincent Lombard, William Helbert, Bernard Henrissat, Nicolas Terrapon

**Affiliations:** 1 AFMB UMR 7257, Aix Marseille Univ, CNRS, Marseille, France; 2 AFMB USC 1408, INRAE, Marseille, France; 3 BBF UMR 1163, INRAE, Marseille, France; 4 CERMAV, Univ. Grenoble Alpes, CNRS, Grenoble, France; 5 Technical University of Denmark, DTU Bioengineering, Kgs Lyngby, Denmark

**Keywords:** Carbohydrate-active enzymes, Protein subfamilies, Sequence similarity networks, Functional annotation, Biochemical characterization, Glycoside hydrolase family 2

## Abstract

**Background:**

Glycoside Hydrolase family 2 (GH2) is one of the largest and most functionally diverse carbohydrate-active enzyme families. This functional diversity is an obstacle to accurate functional prediction by family assignment and has led to the accumulation of erroneous annotations in non-curated databases.

**Results:**

We explored the sequence space of the GH2 family using Sequence-Similarity Networks coupled with closeness centrality to identify 23 subfamilies. The analysis suggests that the GH2 family evolved via multiple duplications followed by neofunctionalization events, with two main activities, β-glucuronidase and β-galacturonidase, re-emerging from likely flexible/reversible ancestors, while an early diverging branch gave birth to several subfamilies with unique activities. To increase the predictive power of subfamily assignments, we biochemically characterized seven members of four of the five subfamilies without previously reported activity.

**Conclusions:**

The GH2 subfamilies showing high functional homogeneity will enable more precise functional predictions, while our work highlights subfamilies that require further biochemical and structural investigations.

**Supplementary Information:**

The online version contains supplementary material available at 10.1186/s13068-025-02669-8.

## Background

Glycoside hydrolases (GHs) have been classified into sequence-based families for more than 30 years [1]. This classification, implemented in the carbohydrate-active enzyme (CAZy) database (www.cazy.org), has demonstrated high predictive power as the catalytic mechanism, catalytic residues and protein fold are conserved within the families [2, 3]. However, sequence-based families often group together enzymes that act on different substrates; therefore, the assignment of a protein to a family does not allow for deduction of a precise functional annotation. This observation has prompted earlier subdivisions of large multifunctional families such as GH13 [4], GH30 [5], GH5 [6], GH43 [7], GH16 [8] and GH31 [9] into subfamilies, which clearly segregate known enzyme activities and thus enable a more precise and reliable assignment of functions in -omics data annotation. With tens of thousands of members on the CAZy website, derived from complete genomes daily released by GenBank, the GH2 family is one of the largest families in the CAZy database [10]. Above 200 members have been experimentally characterized, but 15% were only tested against synthetic substrates. More than ten different activities are referenced (Table 1), with ꞵ-galactosidase dominating.

**Table 1:** Diversity of activities reported in GH2 family

Activity ID	Activity name	EC	Residue −2	Bond −2,−1	Residue −1	Reacting bond	Residue + 1	Bond + 1, + 2	Residue + 2	Characterized
H15	b-Glucosidase	3.2.1.21			[bDGlcp	1,X	Alcohol			3
H17	b-Galactosidase	3.2.1.23			[bDGalp	1,X	Alcohol			112
H19	b-Mannosidase	3.2.1.25			[bDManp	1,X	Alcohol			27
H22	b-Glucuronidase	3.2.1.31			[bDGlcpA	1,X	Alcohol			56
H27	Xylan exo-b-1,4-xylosidase	3.2.1.37			[bDXylp	1,4	bDXylp	1,4	bDXylp	1
H38	b-N-Acetylhexosaminidase	3.2.1.52			[bDGlcpNAc /bDGalpNAc	1,X	Alcohol			1
H41	a-L-Arabinofuranosidase	3.2.1.55			[aLAraf	1,X	Alcohol			2
H85	Lactose b-1,4-galactosidase	3.2.1.108			[bDGalp	1,4	DGlcp]			1
H102	Glycyrrhizin b-glucuronidase	3.2.1.128			[bDGlcpA	1,2	bDGlcpA	1,X	glycyrrhetinic acid	1
H113	Galactan exo-b-1,3-galactosidase	3.2.1.145			[bDGalp	1,3	bDGalp	1,3	bDGalp	1
H114	b-Galactofuranosidase	3.2.1.146			[bDGalf	1,X	Alcohol			6
H119	Mannosyl-oligosaccharide endo-b-1,4-mannosidase	3.2.1.152	[aDManp	1,6	bDManp	1,4	bDGlcpNAc	1,4	bDGlcpNAc	3
H130	Chitosan exo-b-1,4-N-glucosaminase	3.2.1.165			[bDGlcpN	1,4	bDGlcpN	1,4	bDGlcpN	5
H198	a-L-Arabinopyranosidase	3.2.1.-			[aLArap	1,X	Alcohol			3
H212	b-Galacturonidase	3.2.1.-			[bDGalpA	1,X	Alcohol			5
H241	Rhamnogalacturonan II exo-b-1,3-galacturonidase	3.2.1.-			[bDGalpA	1,3	aLRhap	1,3	bDApif	1

CAZac Nomenclature [22] (ID, name) describes the residues and bonds within the catalytic pocket from −2 to + 2. The specificity is mostly held in the reacting bond and residue −1, highlighted in bold blue. Left brackets indicate terminal residues and X unspecified aglycon bonds. Prevalence of characterized members are indicated in the right-hand column

Historically, the most prominent GH2 family members have been the homologs of the LacZ β-galactosidase from *Escherichia coli* [11], which played a key role in the emergence of the operon concept [12]. The human genome encodes two GH2 members, namely the lysosomal β-glucuronidase Gus [13] and β-mannosidase ManbA [14], whose defects lead to severe lysosomal syndromes. GH2 enzymes are characterized by their large size, notably 1023 amino acids for *E. coli* LacZ, originating from a multimodular structure featuring a central (β/α)_8_ catalytic module flanked by four non-catalytic modules [15]. Mechanistically, enzymes of the GH2 family cleave equatorial glycosidic bonds via a classical double displacement mechanism leading to overall retention of the anomeric configuration at the site of cleavage. Catalytic acid/base and nucleophile have been identified by Withers’ laboratory [16].

The extensive characterization of this family was initially fueled by strong industrial interest in β-galactosidase activity, especially in the dairy industry and in the design of galactooligosaccharides as prebiotic agents [17]. The high proportion of β-galactosidases in the family still strongly influences the automatic tools used for genome annotation and this led to the spread of many erroneous annotations that now pollute non-curated databases. A striking example could be found in a work examining the operon that orchestrates rhamnogalacturonan type-II degradation by the human gut symbiont *Bacteroides thetaiotaomicron* [18]. In this study, four GH2-containing proteins with distinct activities were characterized: a β-galactosidase, a β-glucuronidase and two with novel activities for the GH2 family at that time, namely an α-L-arabinopyranosidase and a β-galacturonidase. However, all four proteins are still annotated as β-galactosidases in both GenBank [19] and UniProt [20] protein databanks.

The subdivision of a protein family into more similar subfamilies can be performed via various methods, including phylogenetic reconstructions (requiring the computation of a meaningful multiple sequence alignment) or sequence similarity networks (SSNs; based on pairwise alignments), the latter being more adapted to large and ancient families. Regardless of the method used, an important question concerns the choice of the threshold to define the granularity of the division. Here, we applied an SSN analysis guided by the “closeness centrality” criterion, which was recently exploited to divide six CAZy families into subfamilies as a proof-of-concept [21]. We report the subdivision of the GH2 family into 23 subfamilies, of which 18 harbored experimentally characterized members. Most of these characterized subfamilies display a single function. To facilitate future functional assignments in subfamilies with no characterized members, we recombinantly produced and biochemically determined the substrate specificity of seven members of four previously uncharacterized subfamilies. This work thus provides increased predictive power for this large and diverse enzyme family.

## Methods

### Data preparation

Initially, a total of 65,534 proteins containing GH2 domains were extracted from the CAZy database in July 2023. Catalytic domain sequences were extracted from the full proteins via in-house scripts to ensure at least > 50% coverage of the family profile HMM after using HMMer3.3 [23], and alternatively possible fragments resulting from the inspection of local-alignments of HMMer3.3 searches were reassembled. To reduce the impact of highly similar sequences, a clustering was applied as a redundancy filter to retain only a single representative sequence above a threshold of 80% sequence identity using CD-HIT [24], leading to a final dataset of 9,185 sequences.

### Closeness centrality of SSNs

Domain sequences were subjected to pairwise alignment using Diamond v2.015 [25] with the –ultra-sensitive parameter. For each e-value threshold, from 10^–20^ to 10^–100^ (by step of 10^–1^), an SSN was generated and the weighted average *closeness centrality* of its connected components was computed. Eighty SSNs were thus generated by considering domain sequences as nodes and by creating the list of edges (connections between two nodes) including all pairwise alignments between two domain sequences that obtained an e-value better than the considered threshold. The closeness centrality values were computed for each threshold/SSN using the Python library networkX 2.5 [26] and plotted in R.

### Requirements for subfamily creation

The minimal requirements to create a subfamily were guided by the subfamilies that displayed at least one characterized member. Prior to the novel biochemical characterizations presented in this study, the smallest subfamilies with characterized members contained 34, 35 and 36 members after the redundancy filter (GH2_8, GH2_15 and GH2_22, respectively). Our SSN analysis revealed many smaller groups containing from a single sequence and up to eight groups ranging from 17 to 26 sequences. One of these small groups, with 22 sequences, was characterized in this manuscript and became the GH2_23 subfamily. The seven others will not be released as subfamilies until publication of a biochemical characterization. The released GH2 subfamilies hence include at least either 30 diverse sequences or one characterized member.

### Analysis of secretion signals

Signal peptides were detected using SignalP, TargetP v2 and Phobius [27–29]. Proteins containing transmembrane domains were filtered out using TMHMM [30]. The results were displayed using the R packages ggplot, ggimage, and pheatmap with clustering default options [31–33].

### Structural analyses

Representative structures in subfamilies were extracted from the PDB database [34] when available; otherwise, AlphaFold2 (AF2) models were downloaded directly from the UniProt database [20]. The superposition of structures was performed using the alignment option of PyMOL 2.5 [35] and adjusted manually when necessary, using catalytic amino acids as references.

### Substrates

Synthetic *p*-nitrophenyl glycosides were purchased from the following suppliers: (i) Megazyme (Bray, Ireland) for β-D-galactopyranoside (*p*NP-β-D-Gal*p*), β-D-mannopyranoside (*p*NP-β-D-Man*p*), β-D-galacturopyranoside acid (*p*NP-β-D-Gal*p*A), xylopyranoside (*p*NP-β-D-Xyl*p*), α-L-arabinofuranoside (*p*NP-α-L-Ara*f*), α-L-fucopyranoside (*p*NP-α-L-Fuc*p*); (ii) Merck (Darmstadt, Germany) for β-D-glucuropyranoside acid (*p*NP-β-D-Glc*p*A), β-D-glucopyranoside (*p*NP-β-D-Glc*p*), β-D-fucopyranoside (*p*NP-β-D-Fuc*p*), β-D-ribofuranoside (*p*NP-β-D-Rib*f*), α-L-arabinopyranoside (*p*NP-α-L-Ara*p*) and α-L-rhamnopyranoside (*p*NP-α-L-Rha*p*); Tokyo Chemical Industry (Japan) for N-acetyl-β-D-glucuropyranoside acid (*p*NP-β-D-Glc*p*NAc) and N-acetyl-β-D-galacturopyranoside acid (*p*NP-β-D-Gal*p*NAc); Toronto Research Chemicals (Canada) 6-Sulfo-N-acetyl-β-D-glucuropyranoside acid (*p*NP-β-D-Glc*p*NAc-6S); Biosynth Carbosynth (Berkshire, UK) for *p*NP-β-D-cellobioside; and ThermoFisher (Waltham, Massachusetts, USA) for *o*-nitrophenyl β-D-galactopyranoside *o*NP-β-D-Gal*p*. Disaccharides (β-1,4 galactobiose and β-1,6 galactobiose), xyloglucan (tamarind), arabinogalactan (larch wood) and galactan (potato) were also purchased from Megazyme. Lacto-*N*-tetraose (LNT, β-D-Gal*p*-1,3-β-D-Glc*p*NAc-1,3-β-D-Gal*p*-1,4-D-Glc*p*) and lacto-*N*-neotetraose (LNnT, β-D-Gal*p*-1,4-β-D-Glc*p*NAc-1,3-β-D-Gal*p*-1,4-D-Glc*p*) were kindly provided by DSM-Glycom (Hørsholm, Denmark). Mucin type III sourced from porcine stomach, procured from Sigma-Aldrich (Germany), features polyLacNAc structures comprising repetitive units of β-D-galactopyranosyl-1,4-N-acetyl-D-glucosamine (β-D-Gal*p*-1,4-Glc*p*NAc), where the terminal galactose can be capped with an α-linked sialic acid. The reported sialic acid content ranged from 0.5 to 1.5% of the total glycan composition. The mucin underwent additional processing by dissolution in water, followed by centrifugation at 20,000 × g for 1 h. The resulting supernatant was dialyzed overnight using 12–15 kDa tubing and lyophilized before being used in enzyme reactions at a concentration of 7 g/l.

### Production of recombinant GH2 enzymes

The genes encoding the different GH2 enzymes were codon-optimized for *Escherichia coli* expression and synthesized by the Joint Genome Institute (Walnut Creek, California, USA). The genes were cloned and inserted into the pHTP1 vector (NZYTech, Portugal), where an N-terminal 6xHis-tag and a linker (MGSSHHHHHHSSGPQQGLR) are placed upstream of the GH2-encoding sequences. Competent *E. coli* BL21 (DE3) cells were transformed with the respective plasmids and selected on LB plates supplemented with kanamycin (50 µg/mL). Overnight precultures of the transformed cells were grown at 37 °C in LB medium and used to inoculate culture media at OD 0.05. For *Sd*GH2_14 and *Ct*GH2_23, recombinant protein production was performed at 20 °C in NZY auto-inducible LB medium (NZY Tech, Lisboa, Portugal). *E. coli* cells harboring *Ss*GH2_7, *Zg*GH2_7, *Am*GH2_9, *Bf*GH2_9 and *Bm*GH2_14 plasmids were grown in standard LB medium at 37 °C until OD reached 0.6–0.8, where recombinant protein production was induced with 0.1 mM IPTG at 18 °C. All cultures were incubated overnight. The cells were harvested by centrifugation, and the resulting cell pellet was resuspended in binding buffer (50 mM imidazole, 500 mM NaCl, 20 mM Tris–HCl, pH 7.4) and sonicated using an UP400S Ultrasonic processor (Hielscher Ultrasonics, Teltow, Germany). Recombinant proteins from the supernatants were purified using Ni2 + Sepharose resins (Cytiva, Marlborough, MA, USA) and eluted in a buffer containing 250 mM of imidazole, 500 mM NaCl and 20 mM Tris–HCl at pH7,4. Enzymes were further purified by gel-filtration chromatography, using a HiLoad^™^ 16/600 Superdex^™^ 200 column (Cytiva, Marlborough, Massachusetts, USA). Samples were run at a flow rate of 1 mL min^−1^ in 20 mM Tris–HCl, 100 mM NaCl at pH 7.4. The purified proteins were visualized on SDS-PAGE, and their concentrations were determined by measuring the absorbance at 280 nm and using the respective extinction coefficients and molecular weights of the enzymes predicted by the web server ExPASy ProtParam [36].

### Activity screening against synthetic substrates

The standard conditions for screening activity against 4-nitrophenyl (*p*NP) glycoside substrates were 0.5 mM *p*NP substrate in 50 mM UB4 buffer [37] at pH 5 and 7 with 1 µM purified recombinant enzyme at 35 °C. After 1 h, the enzymatic reaction (100 μL) was stopped by the addition of one volume of 1 M NaCO_3_ (100 μL), and the absorbance values were read at 405 nm. Blank samples were prepared by using heat-inactivated recombinant enzymes, while keeping the other parameters identical. As an additional control for any possible residual activity from native *E. coli* enzymes, post-sonication supernatants from BL21 *E. coli* production strains harboring the same plasmid but with various non-glycoside hydrolase genes were assayed under similar conditions and revealed no activity. All reactions were prepared in triplicates.

### Activity screening against natural glycans

The hydrolytic activities of small oligosaccharides and large polysaccharides were assessed by thin layer chromatography (TLC). Enzymatic reactions were carried out at 35 °C using 4 g.L^−1^ of disaccharides or oligosaccharides and 1.25 μM of the enzyme in 50 mM of UB4 buffer at the optimum pH of the corresponding enzymes. After 16 h of reaction, 1.5 µL of the reaction samples were applied onto a TLC Silica 60 gel (Merck). TLC sheets were then run using a 5:2:4:1 butanol:methanol:ammonium hydroxide:water migration buffer [38]. Products were visualized by spraying a solution containing 2 g diphenylamine, 2 ml aniline, 80 mL methanol, and 10 mL o-phosphonic acid followed by heating.

### Biochemical characterization of the enzymes

#### Thermal unfolding and determination of melting temperatures

The thermal unfolding of the enzymes was determined by following the evolution of the emission of fluorescence at 330 and 350 nm, using a Prometheus Panta (NanoTemper Technologies, Munich, Germany). All samples were prepared in duplicates at 5 µM in 20 mM Tris–HCl buffer, 100 mM NaCl, pH 7.4 and heated from 25 to 90 °C at a rate of 1.5 °C/min. The onset temperature is described as the starting point at which the protein begins to unfold, whereas the melting temperature, Tm, is described at the transition midpoint at which half of the protein population is folded and half of the population is unfolded.

#### pH optimum

The optimum pH was determined by measuring initial rates over 15 min for all the enzymes using *p*NP-Gal*p* as substrate with pH values varying from 3 to 9 and otherwise standard assay conditions.

#### Determination of kinetic parameters

The kinetics parameters were determined in triplicates on *p*NP-β-D-Gal*p* at 40 °C, in a 50 mM buffer, adjusted to the optimum pH of the enzyme, using 1 μM of enzyme and varying substrate concentrations from 0.025 to 14 mM. For each substrate concentration, initial velocities were determined by sampling at regular time intervals when less than 5% of the substrate was consumed. Total reaction time was adapted to each enzyme to capture the initial reaction rate, varying from 5 to 20 min. Reaction samples were stopped by the addition of one volume of 1 M NaCO_3_. The absorbance values were read at 405 nm and converted to mM of *p*NP released using a standard curve. The kinetic parameters (k_cat_, K_M_, k_cat_/K_M_) were calculated using Origin(Pro), Version 2019 (OriginLab Corporation, Northampton, MA, USA) by fitting the Michaelis–Menten model. When substrate saturation could not be reached, k_cat_/K_M_ was estimated using linear regression.

## Results

### Sequence similarity network analysis

**Fig. 1:**
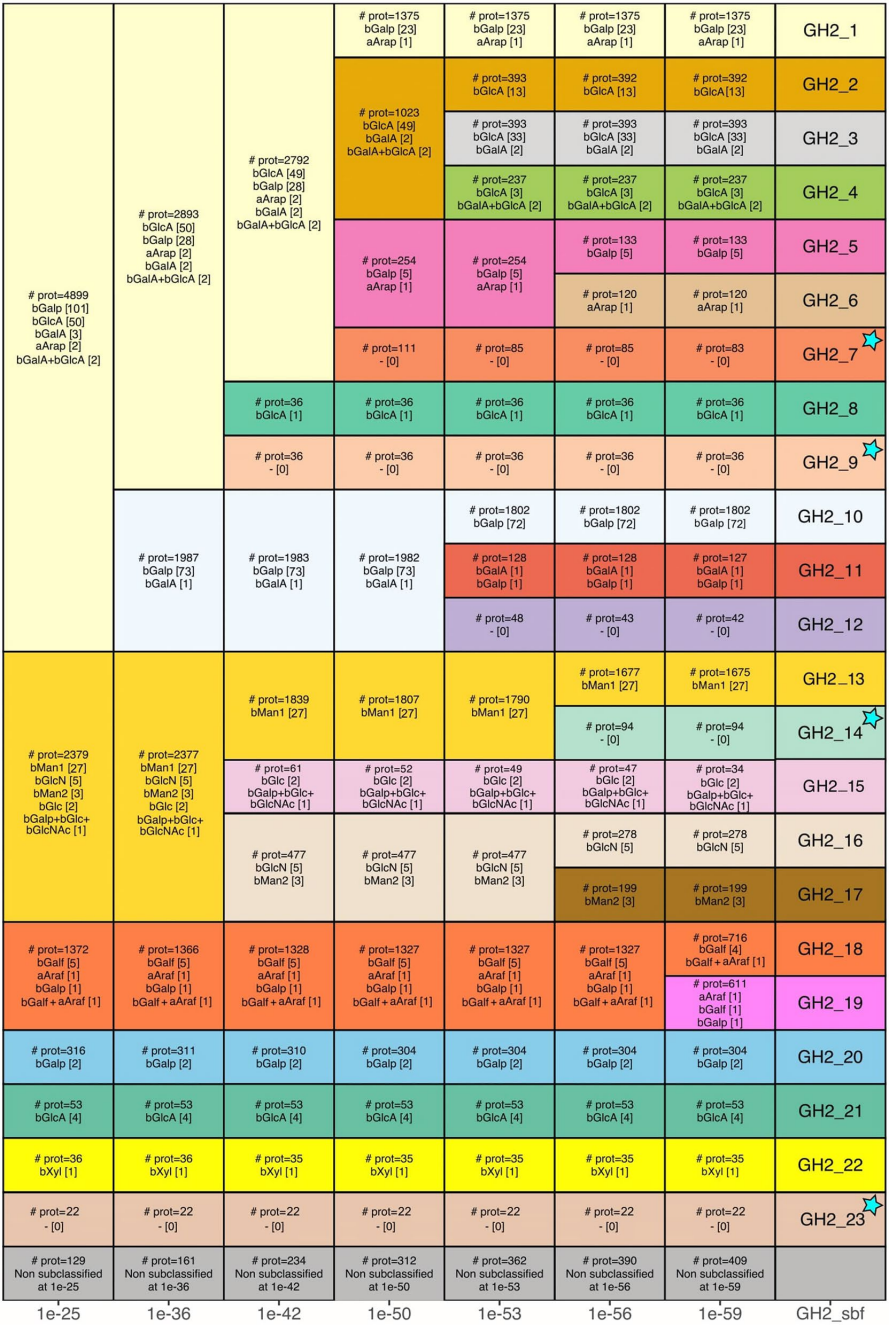
GH2 subfamily emergence along E-value thresholds. From left to right, decreasing E-value thresholds (indicated at the bottom) that correspond to SSN closeness centrality peaks﻿ (Suppl. Fig. S1, Additional File 1), allow the split of the GH2 family into subgroups. For each subgroup, we indicate the number of proteins (label “# prot”) and simplified activities (corresponding to Residue -1 in Table 1, except bMan2 standing for endo-activity H119) with the number of reported characterizations in the CAZy database in square brackets. In rare cases, when several activities are reported for the same protein, we combine the different activities separated by a ‘ + ’ character. The bottom grey row indicates the number of proteins not classified in any subgroup. The final subfamilies are indicated in the right column. The subfamilies having their first characterized member in this work are highlighted by a cyan star

More than 60,000 GH2 domain sequences were extracted from the CAZy database. This dataset was reduced to 9,185 after filtering out the most similar sequences. Pairwise alignments of all the domains allowed the construction of eighty SSNs corresponding to E-value thresholds from 10^–20^ to 10^–100^. Some values were identified as the most relevant options for the subfamily division, based on the *average closeness centrality* criterion (Suppl. Fig. S1, Additional File 1), as previously described [21]. Indeed, local optima are depicted by important increases in this criterion between two consecutive E-value thresholds, hereafter referred to as *peaks*. Each peak reflects the separation of two or more large groups (termed connected components) in the network. These separations indicate major evolutionary events, such as duplications or speciation followed by specialization/conservation and loss of intermediates, in the family history as discussed hereafter and illustrated in Fig. 1. The division of the GH2 family into subfamilies corresponds to the peak at *e*-value 10^–59^ which coincides with the maximal closeness centrality, resulting in subfamilies of high functional homogeneity and only few non-subclassified members. The 23 subfamilies are presented in more detail, including taxonomic distribution, multimodularity, average length, reported activities and solved structures, in Additional File 2.

The first peak, at 10^–25^, is mainly induced by the split of three large groups, hereafter referred to as: *group I,* which encompass subfamilies GH2_1 to GH2_12; *group II* for subfamilies GH2_13 to GH2_17 and *group III* for GH2_18 and GH2_19. This also led to the emergence of the small-to-mid-sized subfamilies GH2_20 to GH2_23.

*Group I* progressively splits into 12 subfamilies, first with the separation of GH2_1 to GH2_9 from GH2_10 to GH2_12, at 10^–36^. GH2_8 and GH2_9 subfamilies both emerge at 10^–42^. At 10^–50^, we observe a separation of: (i) the large GH2_1 subfamily; (ii) the group from GH2_2 to GH2_4; (iii) the GH2_5 and GH2_6 group; and (iv) the small GH2_7 subfamily. At 10^–53^, GH2_2 to GH2_4 individualize into mid-sized subfamilies, simultaneously with GH2_10 to GH2_12. The last split in *group I* occurs at 10^–56^ with the individualization of GH2_5 from GH2_6. *Group II* splits at 10^–42^ into two groups of two subfamilies and the small GH2_15. These four subfamilies individualize at 10^–56^, notably with the large GH2_13. *Group III* remains as a single unit until 10^–59^ where it splits into GH2_18 and GH2_19.

Interestingly, while only sequence-based, this subdivision of the GH2 family shows high functional homogeneity, with most characterized subfamilies displaying a single function. Noteworthy, the most frequently reported activities (> 75%), namely β-galactosidase and β-glucuronidase, are mostly restricted to subfamilies in *group I* but shared across several of these subfamilies, whereas the less frequent activities reported in *group II* appear unique and specific to each of these subfamilies.

The emergence of the unique/specific subfamilies in *group II* occurs at 10^–56^, whereas the final split at 10^–59^ separates GH2_18 from GH2_19 in *group III* which one might question, given the similar activities in these two subfamilies. However, the two subfamilies are well distinguished by their profile HMM, the main bioinformatics annotation tool in CAZy, with only few sequences lying in-between (low increase of non-subclassified, bottom row of Fig. 1). They also show opposite trends in the presence of secretion signals (see hereafter). This split is not due to taxonomy, as both GH2_18 and GH2_19 subfamilies include members of archaeal, bacterial, and fungal origin, with many genomes encoding proteins belonging to these two subfamilies. Conversely, the next peak, at 10^–65^ (data not shown), led to the appearance of four very small subgroups (< 50 members) emerging from subfamilies GH2_2, GH2_3, GH2_13 and GH2_19, while the number of unclassified sequences increase substantially. Consequently, the optimal value of closeness centrality obtained at 10^–59^ was confirmed as the threshold for the division of the GH2 family into 23 subfamilies.

### Secretion signals

We observed important differences in the presence/absence of predicted secretion signals across the GH2 subfamilies. Most of the subfamilies display a high frequency of secretion signals: six subfamilies with > 90% secreted proteins—notably GH2_6 to GH2_9, GH2_11 and GH2_23—and nine others with > 75% secreted proteins. In contrast, four families display < 10% secretion signals—GH2_3, GH2_5, GH2_12 and GH2_19—and two others between 25 and 30%. Interestingly, opposite trends are observed in the most evolutionary and functionally related subfamilies: GH2_19 (~ 10%) *vs.* GH2_18 (~ 80%), GH2_3 (~ 10%) *vs.* GH2_2 and GH2_4 (~ 60% and ~ 80%, resp.), and GH2_5 (6%) *vs.* GH2_6 (~ 90%).

**Fig. 2:**
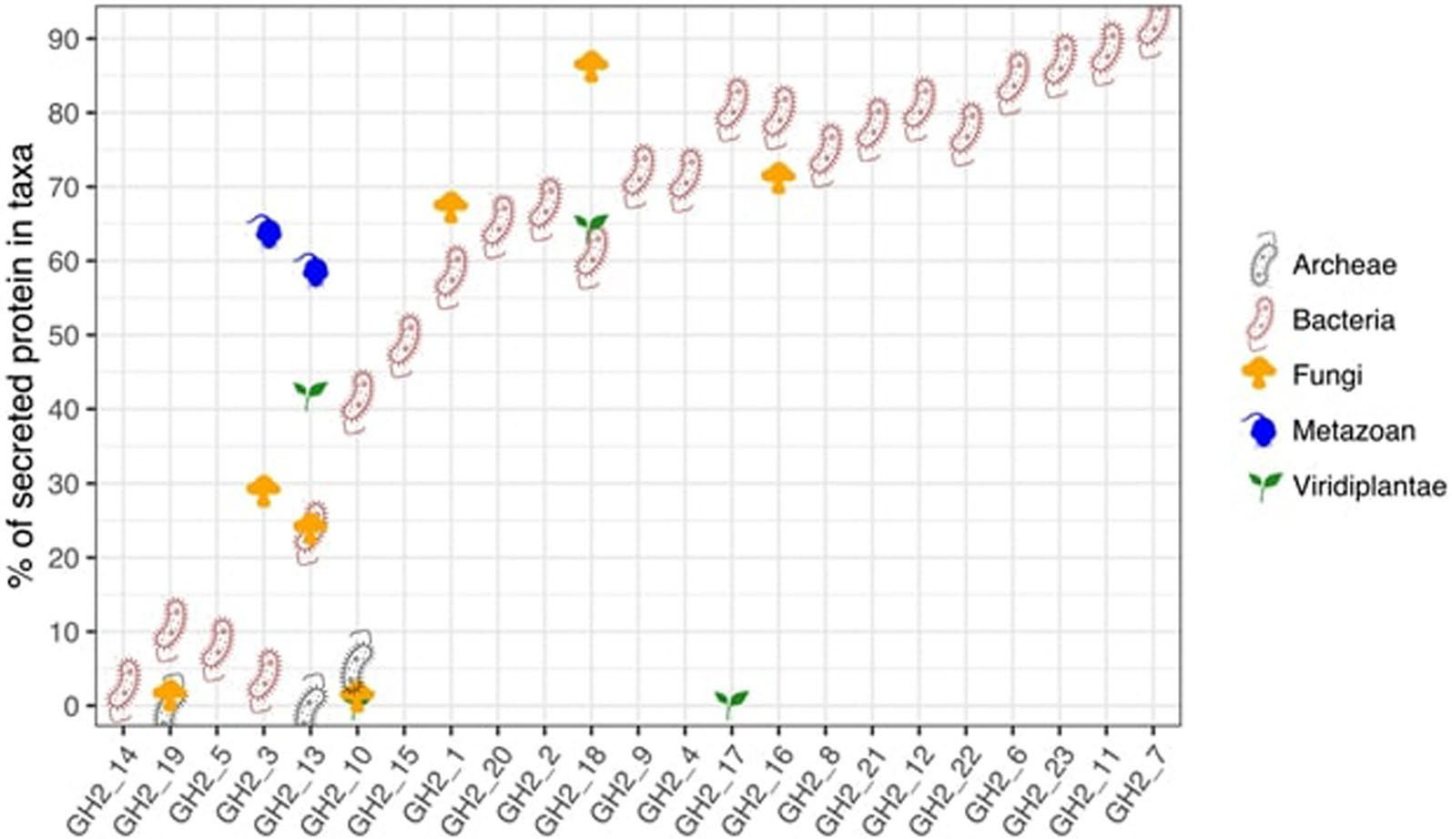
Percentage of secreted GH2 in the major taxonomic groups

We next examined whether the presence/absence of secretion signals was dictated by the taxonomy in several GH2 subfamilies (Fig. 2; Supp. Table S1 and Suppl. Fig. S2, Additional File 1). Metazoan GH2, mostly represented in GH2_3 and GH2_13, were predicted to be secreted more often than other members of these groups. GH2_10 members were predicted to be secreted in ~ 40% of bacterial members but almost never within Fungi and Viridiplantae members. Similarly, almost no secretion signals were identified in Viridiplantae GH2_17 members while most bacterial GH2_17 members were predicted to be secreted. Fungal GH2_1 and GH2_18 members were more often secreted than members of other taxa.

Among Bacteria, the Bacteriodota phylum favors the secretion of GH2 proteins (Suppl. Table S1, Additional File 1). In contrast, only a handful of secreted GH2 proteins were detected in some phyla, such as Bacillota, and none in Spirochaetota and Chloroflexota. Interestingly, Pseudomonadota, Actinomycetota and to a lesser extent Planctomycetota and Verrucomicrobiota display highly contrasting patterns, each taxon having different GH2 subfamilies highly secreted while other not, suggesting that secretion is not necessarily correlated with taxonomy but also likely related to subfamily and function.

### Selection and recombinant production of targets from uncharacterized subfamilies

Seven enzymes from four uncharacterized subfamilies were selected for this study, including: two enzymes from GH2_7 subfamily (*Zg*GH2_7 and *Ss*GH2_7, sharing 36% of identity), two enzymes from GH2_9 subfamily (*Am*GH2_9 and *Bf*GH2_9, sharing 46% of identity), two enzymes from GH2_14 subfamily (*Bm*GH2_14 and *Sd*GH2_14, sharing 38% of identity), and one enzyme from GH2_23 subfamily (*Ct*GH2_23) as shown in Table 2. Both GH2_7 enzymes possess a signal peptide and have been identified in the marine *Zobellia galactanivorans* [39] and the permafrost *Spirosoma spitbergense* [40] bacteria belonging to the Bacteroidota phylum, like the majority of GH2_7 members. *Am*GH2_9 was identified in *Akkermansia muciniphila* [41]*,* a well-known mucin degrader of the Verrucomicrobiota phylum, and *Bf*GH2_9, pertains to *Bacteroides fragilis*, a resident of the human gastrointestinal microbiota [42]. Both possess signal peptides, as most enzymes from the GH2_9 subfamily. Conversely, in the GH2_14 subfamily which has the fewest members with signal peptides, the two selected enzymes, *Bm*GH2_14 from *Burkholderia mallei,* a Betaproteobacteria infectious agent of glanders disease [43], and *Sd*GH2_14 from *Saccharophagus degradans*, a carbohydrate-degrading marine Gammaproteobacteria [44], lack signal peptides. Finally, *Ct*GH2_23, identified in *Ruminiclostridium cellobioparum* subsp. *termitidis* of the Bacillota phylum, isolated from a termite gut [45], does not possess a signal peptide, unlike most of the enzymes in this subfamily.

**Table 2:** List of targets for biochemical studies

Short name	GenBank Accession	Organism	SP
*Zg*GH2_7	CAZ97737.1	*Zobellia galactanivorans* DsiJ^T^	Y
*Ss*GH2_7	WP_020606987.1	*Spirosoma spitsbergense* DSM 19989	Y
*Am*GH2_9	ACD05485.1	*Akkermansia muciniphila* ATCC BAA-835	Y
*Bf*GH2_9	CBW22442.1	*Bacteroides fragilis* 638R	Y
*Bm*GH2_14	AAU48942.1	*Burkholderia mallei* ATCC 23344	N
*Sd*GH2_14	ABD79433.1	*Saccharophagus degradans* 2–40	N
*Ct*GH2_23	EMS73711.1	*Ruminiclostridium cellobioparum subsp. termitidis* CT1112	N

Short names were constructed with the first letters of the organisms encoding the selected enzymes, along with their uncharacterized subfamily. SP indicates the presence (Y) or absence (N) of predicted signal peptides.

The successful production and purification of all the enzymes were achieved, as shown on SDS-PAGE (Suppl. Fig. S3, Additional File 1). Of note, *Bm*GH2_14 underwent proteolytic cleavage to a smaller mass, but the purified fragment retained a high activity, strongly suggesting that the C-terminal non-catalytic modules were cleaved off. This is likely attributed to the large size of the proteins and their multidomain organization. Sufficient quantities of all the enzymes were obtained for further activity screenings and subsequent biochemical characterization.

### Activity screening in subfamilies GH2_7, GH2_9, GH2_14 and GH2_23

The activity screening assays against a panel of different *p*NP-β-D- and *p*NP-α-L-glycosides revealed that all seven enzymes exhibited activity on *p*NP-β-D-galactopyranoside (*p*NP-β-D-Gal*p*), as detailed in Table 3. All the enzymes also displayed activity on *o*NP-β-D-Gal*p* (data not shown), with activity levels comparable to or slightly lower than the one measured on *p*NP-β-D-Gal*p*.

**Table 3:** Biochemical properties and kinetic parameters of the selected GH2 enzymes from uncharacterized subfamilies

Enzymes	Onset T°C (°C)	Tm (°C)	Optimum pH	k_cat_ (min^−1^)	K_M_ (µM)	k_cat_/K_M_ (M^−1^.s^−1^)
*Zg*GH2_7	38.4 ± 0.9	48.6 ± 0.1	6–8	0.8 ± 0.01	67 ± 8.3	204
*Ss*GH2_7	44 ± 0.3	49.1 ± 0.1	7–8	4.6 ± 0.12	93 ± 8	824
*Am*GH2_9	39.8 ± 0.1	53.5 ± 0.1	5	–	–	9 ± 0.5
*Bf*GH2_9	47.1 ± 0.1	59.3 ± 0.1	7	–	–	7 ± 0.04
*Bm*GH2_14	41.6 ± 3.0	48.3 ± 0.1	7–8	2.8 ± 0.06	97 ± 7.9	488
*Sd*GH2_14	40 ± 0.2	46.4 ± 0.1	8	1.2 ± 0.05	64 ± 12.6	306
*Ct*GH2_23	43.4 ± 0.1	52.5 ± 0.1	8	1.4 ± 0.02	40 ± 1.2	586

The optimum pH and kinetic parameters were determined using *p*NP-β-D-Gal*p* as a substrate

All the enzymes were subsequently characterized with *p*NP-β-D-Gal*p*. The onset temperatures of denaturation of the GH2 enzymes ranged from 40 °C for *Am*GH2_9 to 47 °C for *Bf*GH2_9, whereas the Tm values varied from 46 to 59 °C (Table 3). All further enzymatic assays were performed at 40 °C to ensure that the conditions were below the onset temperature of all the enzymes, except for *Zg*GH2_7 where all the experiments were conducted at 35 °C. The optimum pH values were obtained at pH 5 for *Bm*GH2_14, whereas around pH 7 for *Zg*GH2_7, *Ss*GH2_7, *Bf*GH2_9 and *Bm*GH2_14, and at pH 8 for *Sd*GH2_14 and *Ct*GH2_23 (Table 3; Suppl. Fig. S4, Additional File 1).

The hydrolytic activities against various β-D-galactosides with different linkages, structures, and sugar moieties at the + 1 subsite, were investigated. The tested substrates included lactose, β-1,4-galactobiose, β-1,6-galactobiose, lacto-*N*-tetraose (LNT), and lacto-*N*-neotetraose (LNnT). All reactions were analyzed by TLC (Suppl. Fig. S5, Additional File 1). No significant activity was observed for most enzymes except for the two GH2_7 enzymes, which showed weak activity toward β-1,6-galactobiose. Additionally, *Bm*GH2_14 exhibited weak activity against LNT and β-1,6-galactobiose. All enzymes have also been tested on galactose-containing polysaccharides, including xyloglucan, arabinogalactan, galactan and mucin. However, no activity was detected under the assayed conditions. Given that the two GH2_14 enzymes displayed a substrate specificity different from that of the most closely related subfamily, 27 reported β-mannosidases in GH2_13, *Bm*GH2_14 and *Sd*GH2_14 were assayed on mannobiose and mannotriose, but neither showed a detectable activity.

The kinetic parameters for the hydrolysis of *p*NP-Gal*p* were determined, as summarized in Table 3 (for details see Suppl. Fig. S6, Additional File 1). Enzymes from GH2_7, GH2_14 and GH2_23 subfamilies displayed a classical Michaelis–Menten behavior. These enzymes demonstrated slow catalytic rates, with k_cat_ values ranging from 0.8 min^−1^ for *Zg*GH2_7 to 4.6 min^−1^ for *Ss*GH2_7. They displayed low K_M_ values and catalytic efficiencies (k_cat_/K_M_) ranging from 204 to 824 M^−1^.s^−1^. The two enzymes from the GH2_9 subfamily did not reach substrate saturation, hindering the accurate determination of the k_cat_ and K_M_ values. Instead, through linear regression analysis, the value of k_cat_/K_M_ was estimated at 9 and 7 M^−1^.s^−1^ for *Am*GH2_9 and *Bf*GH2_9, respectively, which is approximately 70 times lower than the catalytic efficiency of *Bm*GH2_14.

### Structure–function relationships

**Fig. 3:**
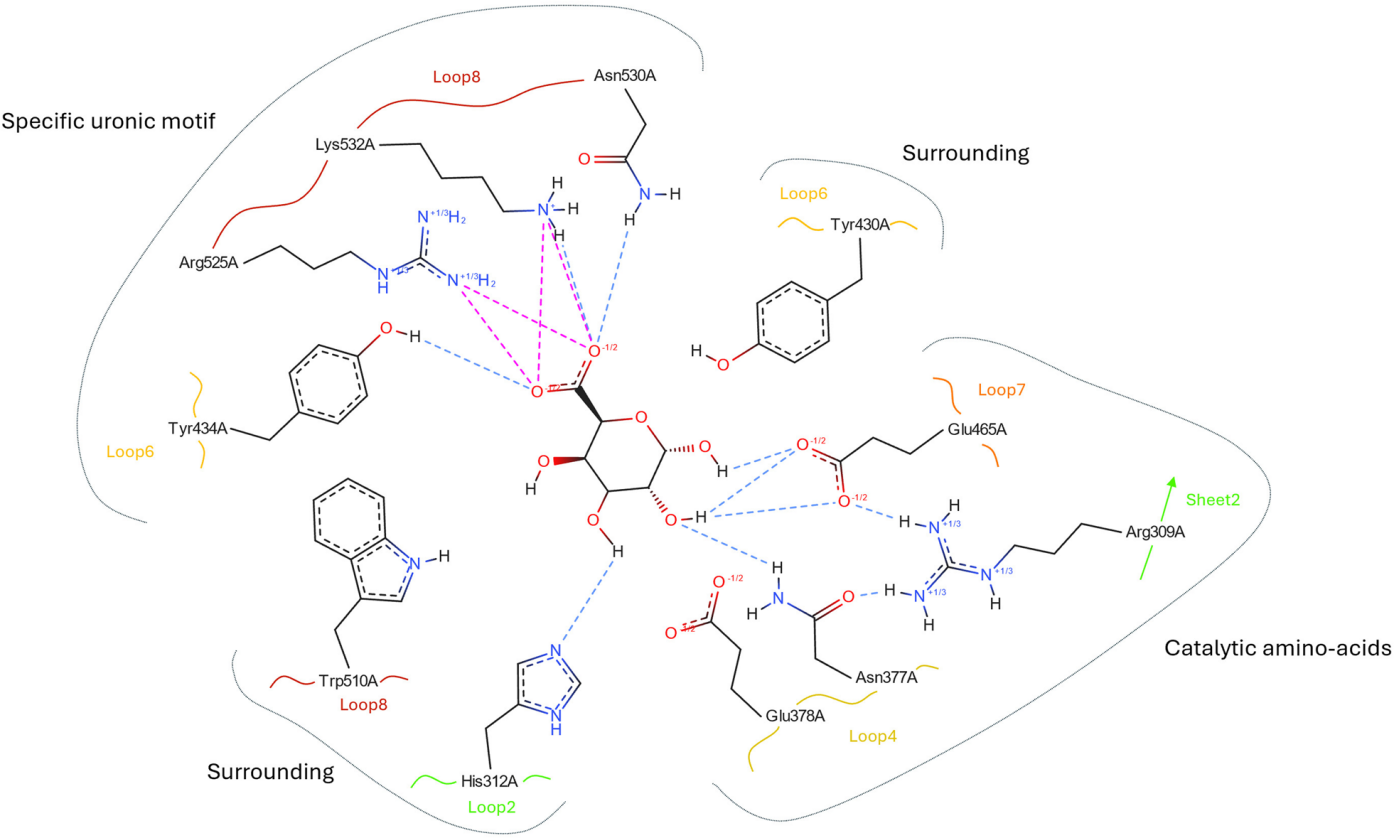
Schematic representation of the active site of the β-galacturonidase from *E. tayi* (6NCX) from the GH2_3 subfamily

To date, 60 GH2 domains possess at least one solved crystal structure (Suppl. Table S2, Additional File 1). The overall architecture of the catalytic domain is the classical (β/α)_8_ barrel of the structural clan GH-A (Suppl. Fig. S7, Additional File 1). GH2 enzymes display a pocket-shaped active site consistent with their exo-activity. The sole exception are the endo-mannosidases in subfamily GH2_17 whose AlphaFold2 (AF2) models show the same restricted pocket but with an opening on the C6 side which would allow for a longer substrate. The superposition of the structures reveals an overall well-conserved architecture of the active site across all the subfamilies, as illustrated in Fig. 3. In particular, the catalytic nucleophile is an invariant Glu in loop 7, assisted by a highly conserved Arg in β-sheet 2. However, specific evolutions of the active site architecture in subfamilies could be related to functional specificities. Substitutions are notably observed in the catalytic acid/base residue, another Glu directly preceded by an Asn (NE motif) in loop 4, as well as three positions surrounding the -1 subsite that limit the size of the catalytic pocket: one His close to the C2/C3 hydroxyl groups (in loop 2), and two aromatics (mainly a Tyr in loop 6 and a Trp in loop 8) that limit the size of the catalytic pocket. These substitutions with known or assumed impacts on specificity are discussed hereafter and summarized in Suppl. Table S3, Additional File 1.

*Group I* is the largest subgroup in terms of the number of sequences and of subfamilies (Fig. 1). It is also the most characterized group, owing to its LacZ [11] and Gus [13] historical enzymes, which belong to GH2_10 and GH2_3 subfamilies, respectively. These two enzymes well illustrate the two main activities of this group: (i) the β-galactosidases in subfamilies GH2_1, GH2_5, GH2_7, GH2_9 and GH2_10 and (ii) the β-hexuronidases (mainly β-glucuronidases, and some β-galacturonidases) in subfamilies GH2_2, GH2_3, GH2_4, and GH2_8. Only one subfamily displays both activities, GH2_11, with one β-galactosidase and one β-galacturonidase characterizations [18]. Previous studies highlighted that GH2 β-hexuronidases distinguish from β-galactosidases by their important and specific NxK motif in loop 8, which stabilizes the carboxyl group at C6 of the substrate [46]. These Asn and Lys residues are directly involved in hydrogen bonds with the carboxyl group, assisted by an upstream Arg in loop 8 and the Tyr in loop 6. These four amino acids are highly conserved among β-hexuronidases on solved structures of the closely related GH2_2 [46], GH2_3 [47] (Suppl. Fig. S8, Additional File 1) and GH2_4 [47], and on the AF2 model of the closest homolog (UniProt ACGA02000046) to the sole characterized GH2_8 [48], as well as in each of these subfamily multiple sequence alignments (MSA). However, the AF2 model (UniProt Q8A925) of the characterized β-galacturonidase in the multifunctional GH2_11 subfamily [48], does not reveal any residue occupying these characteristic positions in *group I* β-hexuronidases, which are also missing in this subfamily MSA. This difference could be linked to the functional versatility of the GH2_11 subfamily and suggests that the β-hexuronidase activity does not rely strictly on the NxK motif. The last activity in *group I*, α -L-arabinopyranosidase, was rarely reported but in distinct subfamilies, namely GH2_1 [49, 50] and GH2_6 [48]. This activity is highly compatible within this group, given the similarity of the α-L-arabinopyranose and β-D-galactopyranose structures, only distinguished by the presence/absence of the C-6 hydroxymethyl group. The three subfamilies not functionally characterized in *group I* prior to this study (GH2_7, GH2_9 and GH2_12) do not display the conserved Arg, Tyr and NxK motifs of β-hexuronidases, which aligns with the β-galactosidase activity displayed here by the four characterized proteins in GH2_7 and GH2_9.

*Group II* is divided into five subfamilies that display activities that are more diverse than those in *group I*, with no β-hexuronidase and only few β-galactosidases in GH2_14 and GH2_15. Interestingly, *group II* gathers all the activities with C2 modification: exo- and endo-β-mannosidases (GH2_13 and GH2_17, resp.), β-N-acetyl-hexosaminidase (GH2_15) and β-glucosaminidases (GH2_16) (Fig. 1). This specificity can be explained by structural analyses, since the architecture of the active site around the C2 and C3 hydroxyls seems to be more divergent in *group II* than in the other groups. First, in solved structures of GH2_16 (PDB 2X09 [51]), the NE motif is substituted by an SD (Suppl. Fig. S9, Additional File 1), which is strictly conserved in the subfamily MSA. The introduction of an Asp allows the opening of the catalytic site to accommodate the C2 amination, whereas the Ser substitution overcomes the biochemical incompatibility between this amination and the side chain of the Asn. The Ser substitution is also observed in subfamily GH2_14 (Suppl. Fig. S10, Additional File 1), where an alternative SE motif replaces the usual NE. The second major difference involves the His which is largely conserved among the other groups but only found in the multifunctional subfamily GH2_15 in group *II*, confirmed by the MSAs. Otherwise, this His is substituted by a Trp for the β-mannosidase subfamilies (GH2_13—PDB 6BYE [52]—and GH2_17), by a Glu for the β-glucosaminidase subfamily (GH2_16—PDB 2X09 [51]), and by a Gly in the GH2_14 subfamily. In the AF2 models of *Bm*GH2_14 and *Sd*GH2_14 (UniProt B2UM41 and E1WVB9, respectively), the substitution by a Gly creates a larger catalytic pocket, which together with the SE alternative motif, may allow the accommodation of substituted substrates.

*Group III* is the smallest group and consists of two subfamilies, namely, GH2_18 and GH2_19. The two subfamilies present similar activities on furanoses, α-L-arabinofuranosidase and β-D-galactofuranosidase, which are specific to *group III*. Recently, one of these enzymes was both structurally characterized (GH2_18; PDB 9J6M [53]) but possible determinants of specificity remain to be determined. Interestingly, the solved structure of an uncharacterized GH2_19 (PDB 7XYR) shows that the NE motif is replaced by TV (Suppl. Fig. S11, Additional File 1). This drastic modification is observed several times in this subfamily, as well as NA or NQ alternatives, even if the NE motif is still the most common, as in all four functionally characterized GH2_19 members. Functional investigations will be necessary to determine whether such modifications of the NE motif result in active enzymes, original mechanisms, or activities. The main difference observed during the structural comparison of *group III* with the other groups showed that the Trp involved in the hydrophobic stacking of the substrate ring at the -1 subsite has been replaced by a Thr and is highly conserved in both subfamilies. The resolution of the structure of an enzyme–substrate complex will be required to determine whether this substitution results from phylogenetic drift or directly contributes to specificity, notably to furanose.

**Fig. 4:**
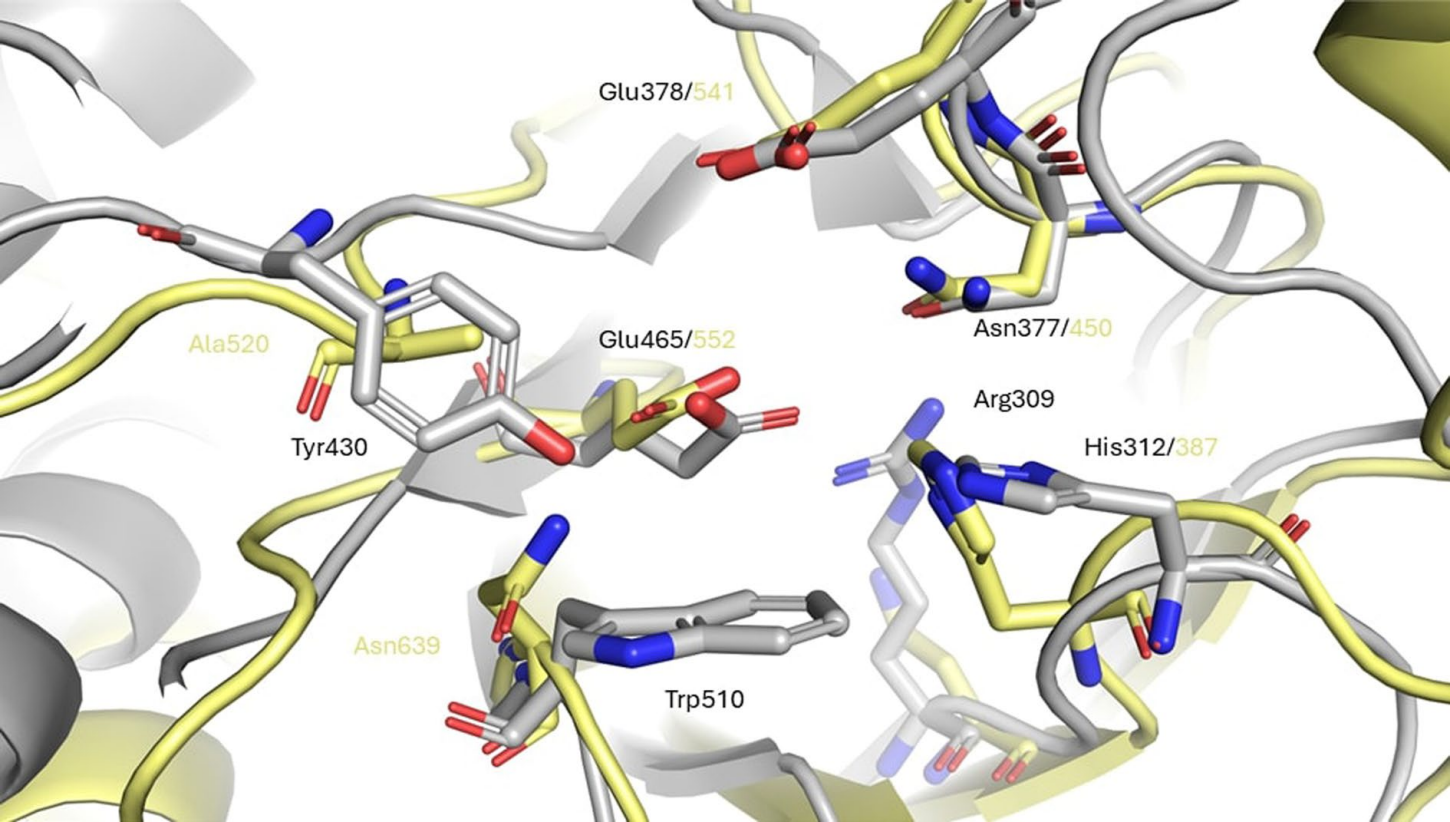
Superposition of the ꞵ-galacturonidase from *E. tayi* (6NCX) in GH2_3 (gray), with the ꞵ-xylosidase from *B. cellulosilyticus WH2* (A0A0P0GUA9) in GH2_22 (yellow)

The four last subfamilies, GH2_20 to GH2_23, emerged first in the SSN analyses. GH2_20 shows β-galactosidase activity [53], but the only solved structure was deposited by a structural genomic consortium (PDB 8U01) without associated publication (Suppl. Fig. S12, Additional File 1). In the absence of biochemical data, we can only extrapolate from the comparison between this structure and the AF2 models of the two functionally characterized members. All three structures lack the aromatic residues that close the active site pocket. The loop 6, carrying the Tyr, is longer in GH2_20 which implies a partial disorganization of helix 8 compared with the structures of the other subfamilies, and no equivalent amino acid replaces it. The Trp (loop 8) is substituted by a shorter hydrophobic amino acid, Leu. This deeper active site is extended by a long groove, which could explain the reported activities on decorated substrates such as xyloglucan or type II rhamnogalacturonan [54, 55]. GH2_21 is the only subfamily outside *group I* with β-glucuronidase activity. It has been reported that the stabilization of the carboxylic group does not involve the NxK motif (as observed in GH2_11) and that the acid/base Glu is replaced by a Trp [56]. The MSA of the subfamily confirms the frequent substitution of the acid/base in this subfamily by other hydrophilic residues (Lys, Arg, or Asn), while another Glu, identified close to the catalytic site and hypothesized to replace the “classical” acid/base [56], is highly conserved in subfamily GH2_21 (E402 in Uniprot T2KN75). Subfamily GH2_22 has the only β-xylosidase activity reported in family GH2 [54]. The AF2 model of the characterized β-xylosidase in GH2_22 (UniProt A0A0P0GUA9) shows conservation of the positions of the catalytic amino acids, the nucleophile, and the NE motif, as well as the His close to the C2/C3 hydroxyl group while the remainder of the active site is quite divergent. The AF2 model shows a substitution of the Arg, which is strictly conserved in the other subfamilies, by an Asp, and the MSA confirms this substitution is evolutionary conserved. Moreover, the couple of aromatics limiting the pocket are not found either, Tyr and Trp are substituted by Ala and Asn, respectively (Fig. 4). In the last and distant GH2_23 subfamily, the member characterized here as β-galactosidase shows all the makers previously described: the superposition of its AF2 model (Uniprot S0FTQ0) with β-galactosidases from *group I*, allows the identification of the conserved catalytic amino acids, the two aromatics and the His involved in C2/C3 limitation. These markers are conserved throughout the whole GH2_23 subfamily despite their distant sequence relatedness to the other groups.

## Discussion

### GH2 subfamilies reflect evolutionary/functional pressures

Proteins encoding a catalytic GH2 domain form a large and diverse family with tens of thousands of members reported in the CAZy database, which are widely distributed across the tree of life. With more than 200 functionally characterized members, and a dozen distinct activities, accurate functional annotation of a GH2 member based on sequence alone is challenging, and erroneous β-galactosidase annotations have contaminated non-curated databases. To improve annotations, a subfamily level classification can provide a solution. We performed a Sequence Similarity Network analysis, which does not suffer from the limitations of multiple sequence alignments and phylogenetic reconstructions, although it cannot capture ancestral relationships such as classical phylogeny. We then measured *closeness centrality* to guide the choice of the optimal division. Our GH2 subclassification assigned 97% of the GH2 members to one of its 23 subfamilies, only five of which initially did not possess any functionally characterized member. We conducted a functional screening of four of those uncharacterized subfamilies and obtained an activity for seven members. Importantly, 14 of the 22 characterized subfamilies display a single activity to date. Among subfamilies with several activities (no more than three), one activity might appear more frequent (e.g., in GH2_1 and GH2_3) while frequently those activities are closely related, suggesting functional promiscuity, such as on α-L-arabinopyranose and β-D-galactopyranose which differ only by the presence/absence of the C-6 hydroxymethyl group. Overall, these results support the use of subfamilies to increase the accuracy of GH2 functional annotations.

Interestingly, five subfamilies (mostly from *group II*) display an activity not found in any other subfamilies, while other subfamilies (mostly from *group I*) share the same and main activities in the family. The β-galactosidases and β-glucuronidases in *group I* do not originate from a single ancestor but rather emerged several times during GH2 evolution, also supported by the initial segregation of such activities in subfamilies GH2_20, GH2_21 and GH2_23. A hypothesis would be an evolutionary scenario in which organisms maintained some GH2 sequences with such ambiguity/promiscuity between β-galactosidase and β-glucuronidase activities. This would have allowed rapid escape from environmental pressures, while other duplications could lead to the specialization of newly created paralogs (while maintaining flexibility in the original copy). These specializations induced either the emergence of unique activities or most frequently to the repeated reappearance of the two main ancestral activities. Similar trends were observed during the recent subclassification of GH16 [8] and GH31 [9].

### Future functional prospections will be essential in small and under-characterized subfamilies

Importantly, the functional specificity of the GH2 subfamilies must be considered in light of the current knowledge, or lack of, notably due to biases in the assayed substrates or in the sequence/taxonomic diversity in each subfamily.

The substrate bias is particularly prominent in GH2 as 15% of characterized GH2 have demonstrated activities limited to synthetic substrates only. Similarly, we failed to identify the natural substrate of the four subfamilies characterized here, nor did we obtain any strong activity on lactose, on the few commercially available oligosaccharides, or on the polysaccharides containing a terminal β-D-galactose. The most important limitation is probably the lack of commercially available substrates, such as agarobiose or end-products derived from the partial degradation of (arabino)galactans. Indeed, both our GH2_7 enzymes appear in Polysaccharide Utilization Loci (PULs [57]) along with GH117 neo-oligo-agarases, while many GH2_9 members co-occur in PULs with GH43 and GH51 α-L-arabinofuranosidases as well as GH95 α-L-fucosidases, and might thus target fucosylated arabinogalactans, which could not be assayed here.

Taxonomic/sequence bias is also frequently underestimated, as characterized members can represent only a reduced sample of the subfamily diversity. For example, in subfamily GH2_15, the characterized members come from a single genus in the Deinococcota phylum [58–60], while 90% of the subfamily are from Gammaproteobacteria. Similarly, the three characterized GH2_17 members belong to plant genomes [61–63] while none are from the large bacterial majority. For more reliable functional annotations, confirming the apparent subfamily specificity will be essential.

Even in *well-characterized* subfamilies, where one activity largely dominates, rare and original activities could be observed, supporting the lesson that “one should expect the unexpected” and reminding that the propagation of annotation based only on “majority-rule predictions” should be avoided. For example, in GH2_1, two α-L-arabinopyranosidases were reported [43, 50] in addition to 23 β-galactosidases, and in GH2_3, two β-galacturonidases [46] were found aside 33 β-glucuronidases. These enzymes indeed cleave very similar but not identical glycosides.

Another important point is to highlight the poorly characterized regions of the GH2 sequence space, with 16 subfamilies having fewer than five characterized members, one subfamily remaining to be characterized, and some small uncharacterized groups that did not fit in any of the 23 subfamilies described here, but that might seed future additional subfamilies after an enzymatic characterization (and/or an increase of their sequence diversity).

Overall, we observe that the division of a carbohydrate-active enzyme family into subgroups often but not always results in one specific activity in a single subfamily owing to the plasticity of the active sites and structural similarities between certain carbohydrate structures. Evolution can reinvent/reproduce the substitutions that shape the catalytic sites and these precise changes escape observations based on sequence only, while fine structural comparisons of the catalytic sites are promising but still challenging.

## Supplementary Information

Additional file 1.

Additional file 2.

## Data Availability

GH2 subfamilies are accessible on CAZy website (www.cazy.org)
