# Supplementary material for: Division of the large and multifunctional glycoside hydrolase family 2: high functional specificity and biochemical assays in the uncharacterized subfamilies
Source: Biotechnol Biofuels Bioprod. 2025 Jul 9;18:68. doi: 10.1186/s13068-025-02669-8 (PMC12243196; doi:10.1186/s13068-025-02669-8)
Supplement: Supplementary file 1 — Additional file 1. [file 13068_2025_2669_MOESM1_ESM.docx]

Supplementary Tables and Figures

**Supplementary Table S1. Secretion signals by taxonomy**. Number of secreted CAZymes in the main taxonomic group for each GH2 subfamily after redundancy filtering.


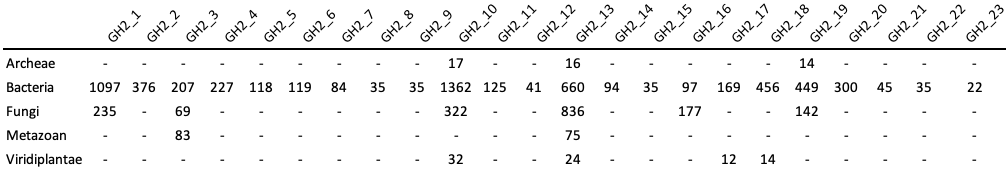


**Supplementary Table S2. GH2 subfamily members with a characterized representative and/or a solved structure in the Protein Data Bank.** A protein with several solved structures (PDB codes) was accounted for only once.

| **Groups** | **Subfamilies** | **EC** | **Residue -1** | **Characterized** | **PDB** |
| --- | --- | --- | --- | --- | --- |
| Group I | GH2_1 | 3.2.1.23 | bDGalp | 23 | 7 |
|  |  | 3.2.1.108 | bDGalp | 1 |  |
|  |  | 3.2.1.- | aLArap | 2 |  |
|  |  | no activity published |  |  | 2 |
|  | GH2_2 | 3.2.1.31 | bDGlcpA | 13 | 9 |
|  |  | no activity published |  |  | 2 |
|  | GH2_3 | 3.2.1.31 | bDGlcpA | 33 | 14 |
|  |  | 3.2.1.128 | bDGclpA | 1 |  |
|  |  | 3.2.1.- | bDGalpA | 2 | 1 |
|  |  | no activity published |  |  | 1 |
|  | GH2_4 | 3.2.1.31 | bDGlcpA | 5 | 4 |
|  |  | 3.2.1.- | bDGalpA | 3† | 2† |
|  | GH2_5 | 3.2.1.23 | bDGalp | 5 | 1 |
|  | GH2_6 | 3.2.1.- | aLArap | 1 |  |
|  | GH2_7* | 3.2.1.23 | bDGalp | 2 |  |
|  | GH2_8 | 3.2.1.31 | bDGlcpA | 1 |  |
|  | GH2_9* | 3.2.1.23 | bDGalp | 2 |  |
|  | GH2_10 | 3.2.1.23 | bDGalp | 76 | 5 |
|  |  | no activity published |  |  | 3 |
|  | GH2_11 | 3.2.1.23 | bDGalp | 1 |  |
|  |  | 3.2.1.- | bDGalpA | 1 |  |
|  | GH2_12 |  |  |  |  |
| Group II | GH2_13 | 3.2.1.25 | bDManp | 27 | 5 |
|  | GH2_14* | 3.2.1.23 | bDGalp | 2 |  |
|  | GH2_15 | 3.2.1.21 | bDGlcp | 3 |  |
|  |  | 3.2.1.52 | bDGxxpNAc | 1† |  |
|  |  | 3.2.1.23 | bDGalp | 2† |  |
|  | GH2_16 | 3.2.1.165 | bDGlcpNAc | 4 | 1 |
|  | GH2_17 | 3.2.1.152 | bDManp | 3 |  |
| Group III | GH2_18 | 3.2.1.146 | bDGalf | 5 | 1 |
|  |  | 3.2.1.55 | aLAraf | 1 |  |
|  |  | no activity published |  |  | 1 |
|  | GH2_19 | 3.2.1.23 | bDGalp | 2 |  |
|  |  | 3.2.1.146 | bDGalf | 1 |  |
|  |  | 3.2.1.145 | bDGalp | 1 |  |
|  |  | 3.2.1.55 | aLAraf | 1 |  |
|  |  | no activity published |  |  | 1 |
| Others | GH2_20 | 3.2.1.21 | bDGlcp | 1 |  |
|  |  | 3.2.1.23 | bDGalp | 1 |  |
|  |  | no activity published |  |  | 1 |
|  | GH2_21 | 3.2.1.31 | bDGlcpA | 4 | 1 |
|  | GH2_22 | 3.2.1.37 | bDXylp | 1 |  |
|  | GH2_23* | 3.2.1.23 | bDGalp | 1 |  |

Presence of a multifunctional enzyme is indicated by a red dagger (†) and novel characterizations realized in this study by a blue asterisk (*).

**Supplementary Table S3. Comparative analysis of the key positions in the catalytic pocket**. For each subfamily, a representative member was selected, and indicated by its activity and PDB/AF2 identifier (except for GH2_8, having no precomputed AF2 model in Uniprot due to length issues: its AF2 model was then locally computed on the first 1132 amino-acids only). The key positions presented in the schematic representation (Figure 3 in the main text) are compared and represented in blue if substituted to a similar amino-acid, or in red for amino-acid of different physico-chemical properties. The uronidases are highlighted with a green background, while the absence of a residue at a position is depicted by a light grey background. GH2_12 Subfamily was not integrated in this analysis due to the absence of both a demonstrated activity and solved structure (in dark grey).

| Groups | Subfamilies | Activities | PDB or AF2 identifier | Catalytic amino acids | | | | Surrounding aromatic | | | Uronic specific motif | | |
| --- | --- | --- | --- | --- | --- | --- | --- | --- | --- | --- | --- | --- | --- |
|  |  |  |  | Nucleophile | Motif incl. acid/base | | Assist |  |  |  |  |  |  |
|  |  |  |  | E | N | E | R | H | Y | W | Y | R | NxK |
| Group I | GH2_1 | ꞵ-galactosidase | 5T98 | E519 | N441 | E442 | R371 | H374 | Y498 | W578 |  |  |  |
|  | GH2_2 | ꞵ-glucuronidase | 6D50 | E526 | N425 | E426 | R360 | H363 | Y495 | W573 | Y499 | R583 | N591xK593 |
|  | GH2_3 | ꞵ-galacturonidase | 6NCX | E465 | N377 | E378 | R309 | H312 | Y430 | W510 | Y434 | R525 | N530xK532 |
|  | GH2_4 | ꞵ-glucos/galacturonidase | 6NCZ | E491 | N406 | E407 | R340 | H343 | Y463 | W536 | Y467 | R546 | N555xK557 |
|  | GH2_5 | ꞵ-galactosidase | 5EUV | E446 | N364 | E365 | R299 | H302 | F410 | W489 |  |  |  |
|  | GH2_6 | α-L-arabinopyranosidase | Q8A934 | E511 | N446 | E447 | R380 | H383 |  | W564 |  |  |  |
|  | GH2_7* | ꞵ-galactosidase | G0L8P5 | E653 | N571 | E572 | R504 | H507 | Y627 | W691 |  |  |  |
|  | GH2_8 | ꞵ-galacturonidase | UVQ63317.1 | E843 | N772 | E773 | R708 | H711 | W818 | W894 | Y822 | R904 | N921xK923 |
|  | GH2_9* | ꞵ-galactosidase | E1WVB9 | E515 | N438 | E439 | R356 | H359 |  | W552 |  |  |  |
|  | GH2_10 | ꞵ-galactosidase | 1YQ2 | E521 | N441 | E442 | R366 | H369 | Y483 | W552 |  |  |  |
|  | GH2_11 | ꞵ-galacturonidase | Q8A925 | E469 | N412 | E413 | R351 | H354 | Y449 | W502 |  |  |  |
|  | GH2_12 |  |  |  |  |  |  |  |  |  |  |  |  |
| Group II | GH2_13 | ꞵ-mannosidase | 6BYE | E575 | N476 | E477 | R408 | W410 | W552 | W667 |  |  |  |
|  | GH2_14* | ꞵ-galactosidase | Q21PE6 | E499 | S417 | E418 | R348 | G351 | Y477 | W601 |  |  |  |
|  | GH2_15 | ꞵ-glucosaminidase | Q745T5 | E436 | N377 | E378 | R312 | H314 | Y543 | F521 |  |  |  |
|  | GH2_16 | ꞵ-glucosaminase | 2X09 | E541 | S468 | D469 | R392 | E394 | Y516 | W642 |  |  |  |
|  | GH2_17 | ꞵ-mannosidase | Q75W54 | E549 | N463 | E464 | R387 | W389 | Y528 | W654 |  |  |  |
| Group III | GH2_18 | ꞵ-galactofuranosidase | 9J6M | E530 | N463 | E464 | R399 | H401 | Y512 | T586 |  |  |  |
|  | GH2_19 | no activity | 7XYR | E498 | T418 | V419 | R353 | H355 | Y469 | T549 |  |  |  |
| Others | GH2_20 | no activity | 8U01 | E501 | N415 | E416 | R346 | H348 | ≠ loop posit. | L576 |  |  |  |
|  | GH2_21 | ꞵ-glucuronidase | 6HPD | E509 | N446 | W447 | R382 | H385 |  | W571 |  |  |  |
|  | GH2_22 | ꞵ-xylosidase | A0A0P0GUA9 | E552 | N450 | E451 | N385 | H387 | A520 | N639 |  |  |  |
|  | GH2_23* | ꞵ-galactosidase | S0FTQ0 | E493 | N417 | E418 | R349 | H351 | Y476 | F545 |  |  |  |


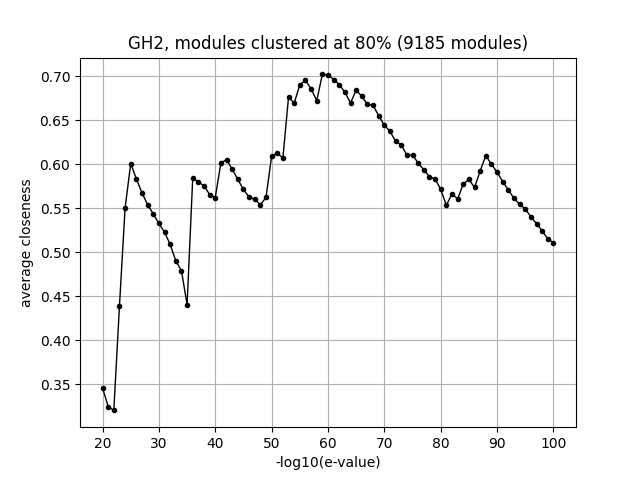


**Supplementary Figure S1 Average closeness centrality in GH2 SSNs**. For each E-value threshold, from 10^-20^ to 10^-100^ by steps of 10^-1^, the list of module pairs (obtaining a significant E-value in the pairwise alignment) was used to compute the average closeness centrality value plotted here. In such a plot, peaks indicate the split of independent subnetworks (connected components), and thus relevant options for the division of the family into subfamilies.


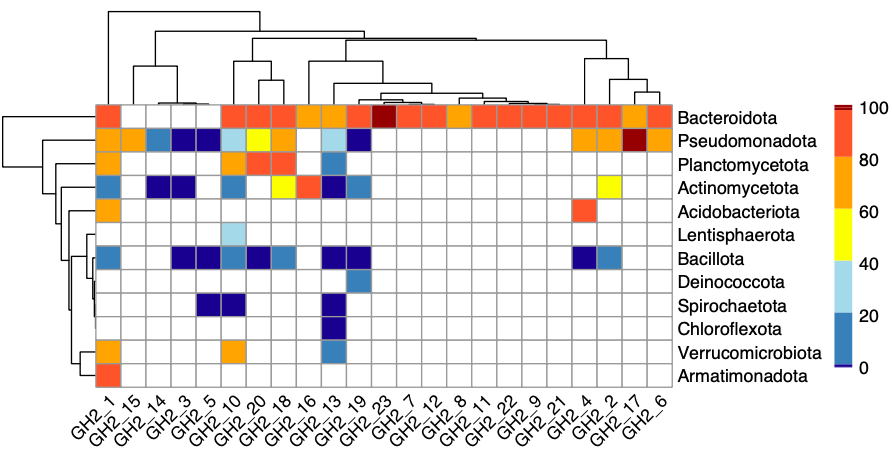


**Supplementary Figure S2. Percentage of signal peptides in each bacterial phylum.** In white, were not considered cases with less than 10 GH2 members encoded by the phylum.


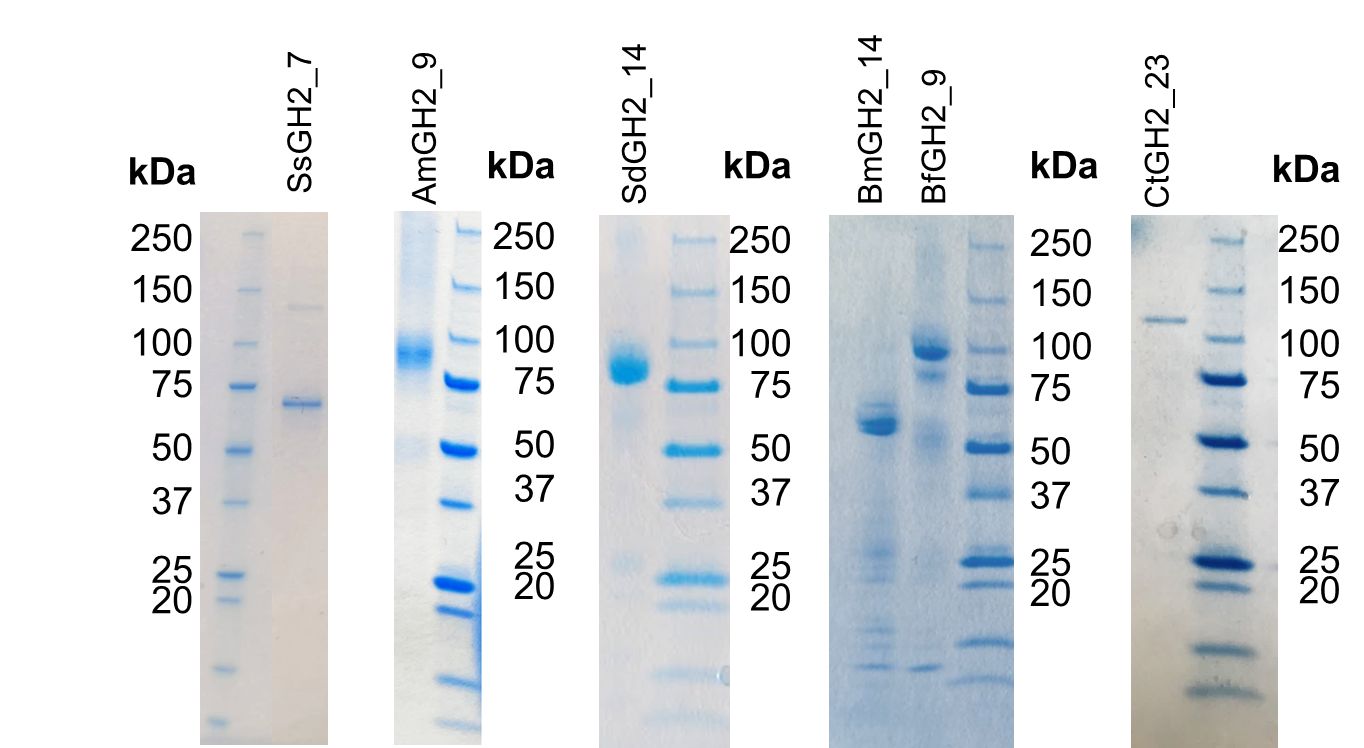


**Supplementary Figure S3. SDS-PAGE of the produced and purified enzymes.** The calculated molecular weights of the recombinant enzymes were of 77.26 kDa for *Ss*GH2_7, 95.02 kDa for *Am*GH2_9, 93.86 kDa for *Sd*GH2_14, 92.90 kDa for *Bm*GH2_14, 96.73 kDa for *Bf*GH2_9, and 129.67 kDa for *Ct*GH_23.


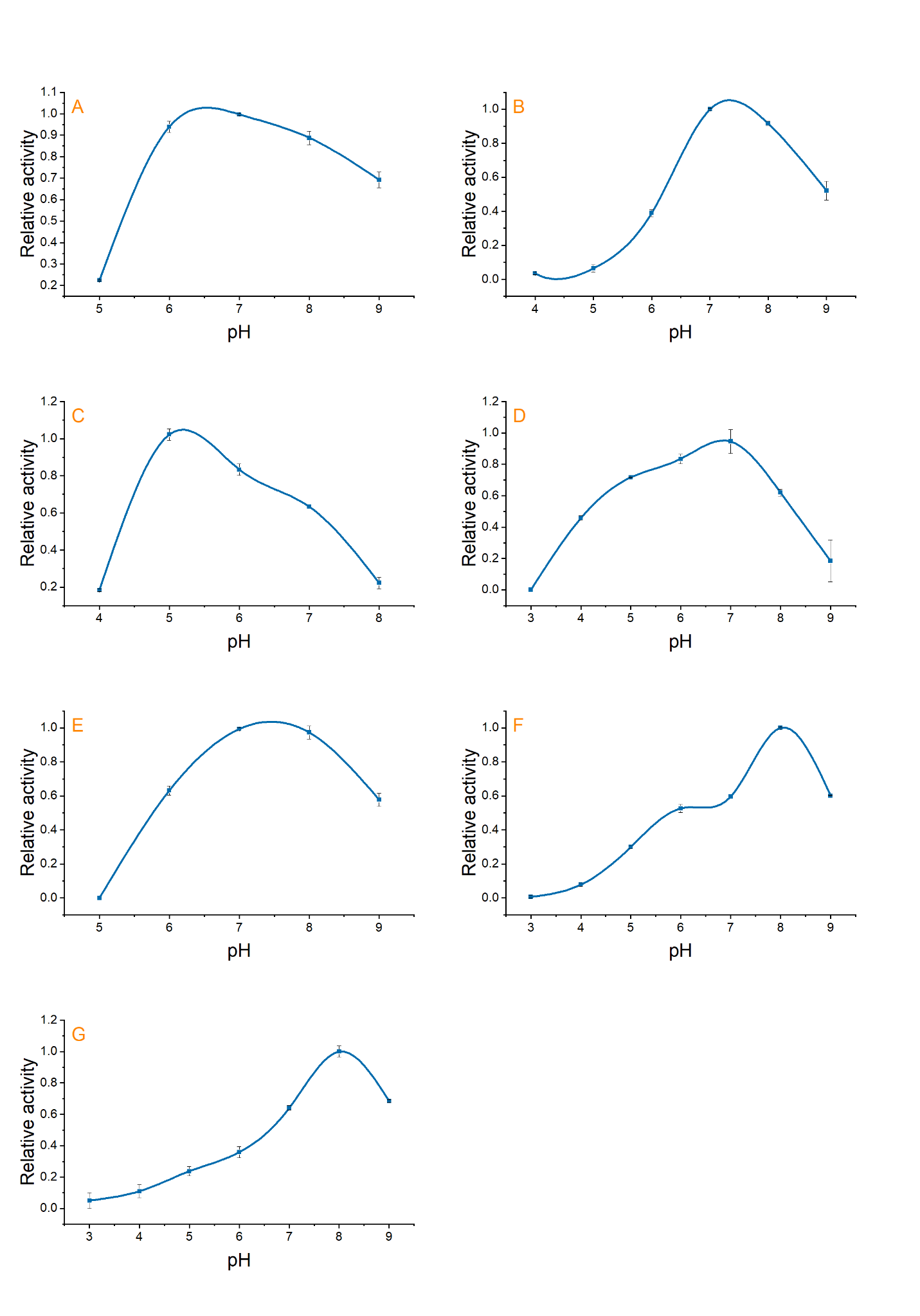


**Supplementary Figure S4. pH optimum of the target GH2 enzymes.** Measurements realized for all enzymes using pNP-Gal*p* as substrate under standard assay conditions in a 50 mM UB4 buffer with pH value varying from 3 to 9. (A) *Zg*GH2_7A; (B) *Ss*GH2_7; (C) *Am*GH2_9; (D) *Bf*GH2_9; (E) *Bm*GH2_14; (F) *Sd*GH2_14; and (G) *Ct*GH2_23


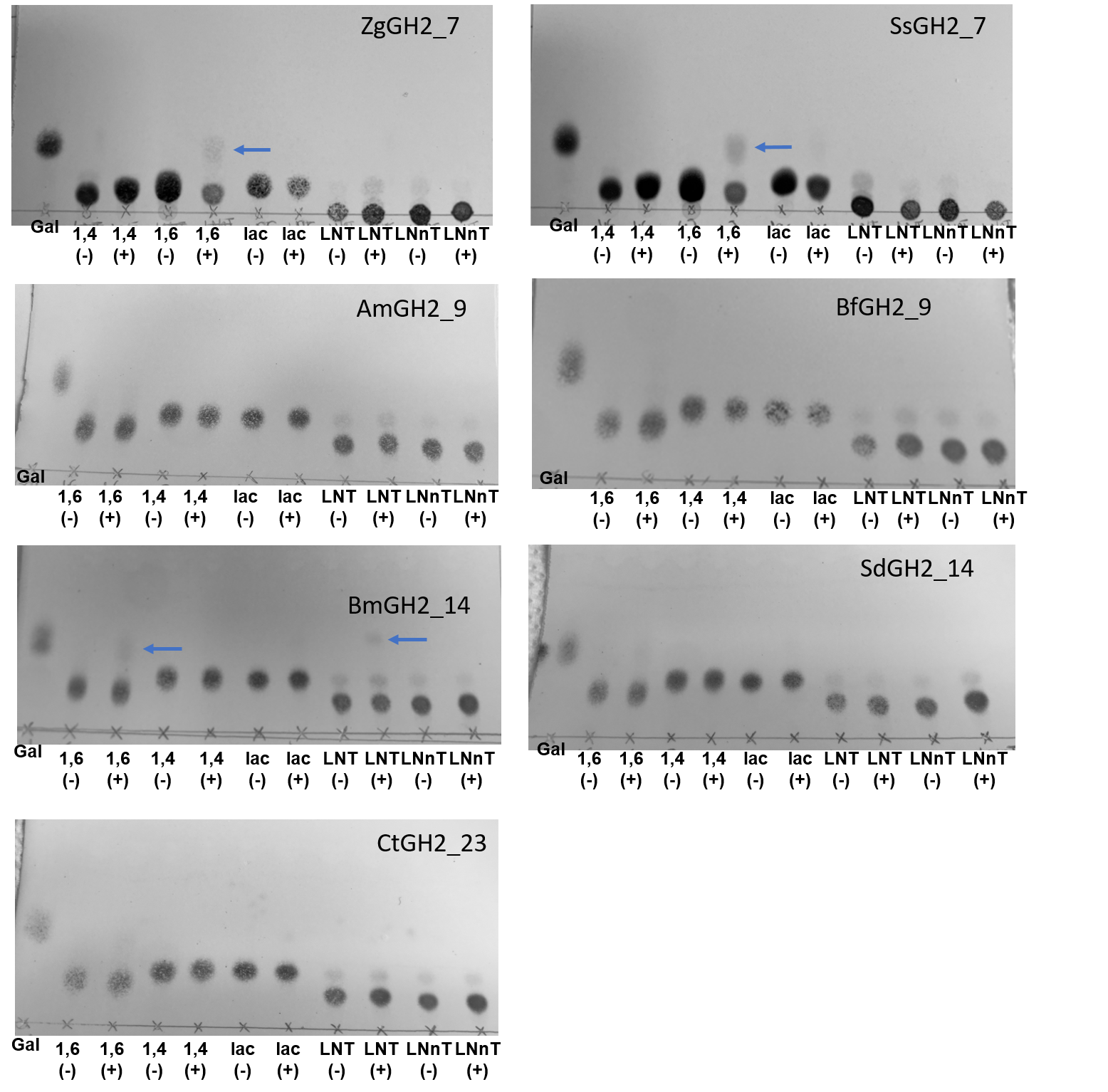


**Supplementary Figure S5. TLC analysis of 16 h reaction on different substrates**. Gal, galactose standard; 1,6, β-1,6 galactobiose; 1,4, β−1,4 galactobiose; lac, lactose. (-) represents the negative control and (+), the 16h reaction with the enzyme.


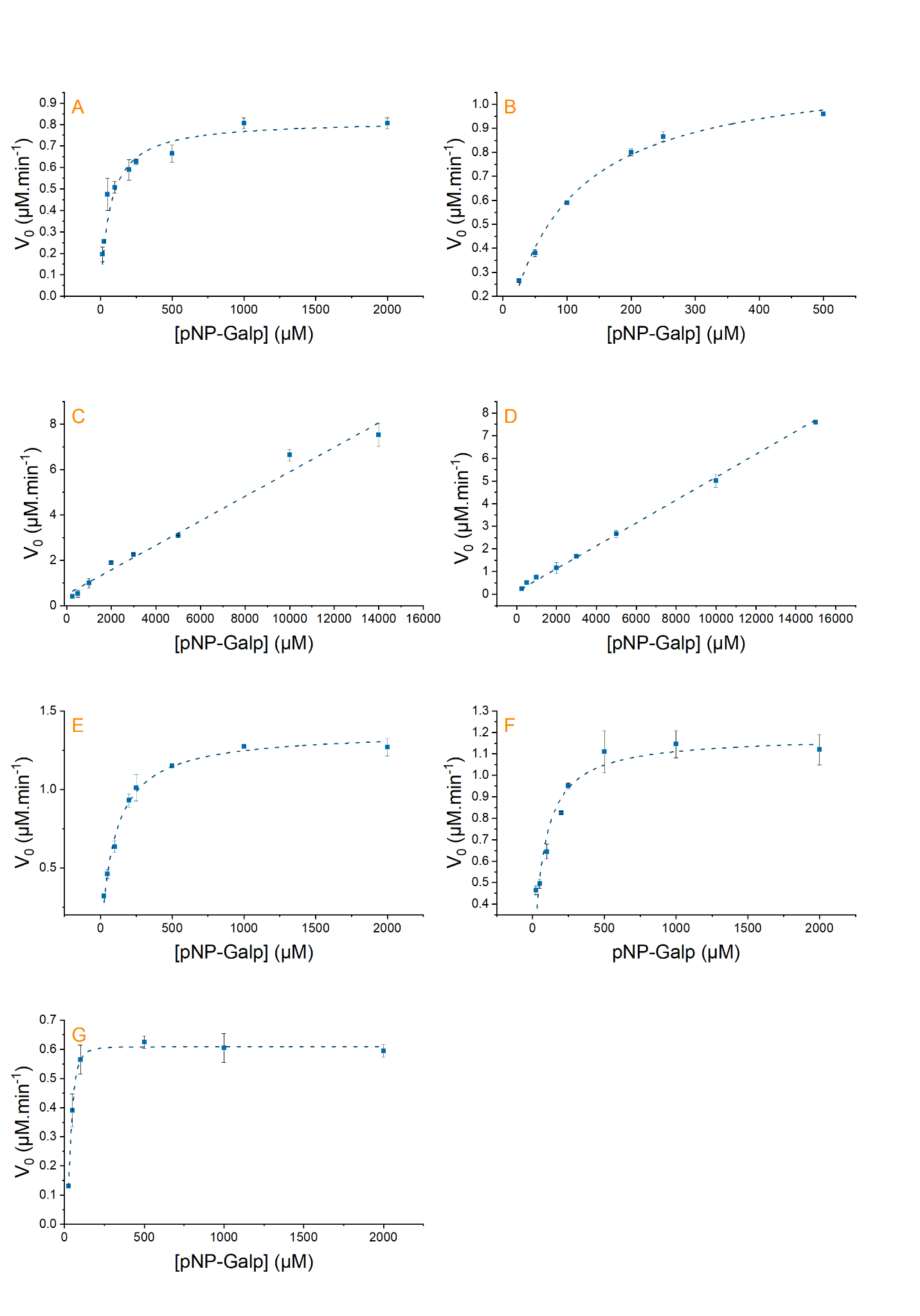


**Supplementary Figure S6. Determination of kinetic parameters on *p*NP-β-D-Gal*p*.** (A) *Zg*GH2_7A (R2=0,950); (B) *Ss*GH2_7 (R2=0,993); (C) *Am*GH2_9 (R2=0,975); (D) *Bf*GH2_9 (R2=0,998); (E) *Bm*GH2_14 (R2=0,990); (F) *Sd*GH2_14 (R2=0,927); and (G) *Ct*GH2_23 (R2=0,996).


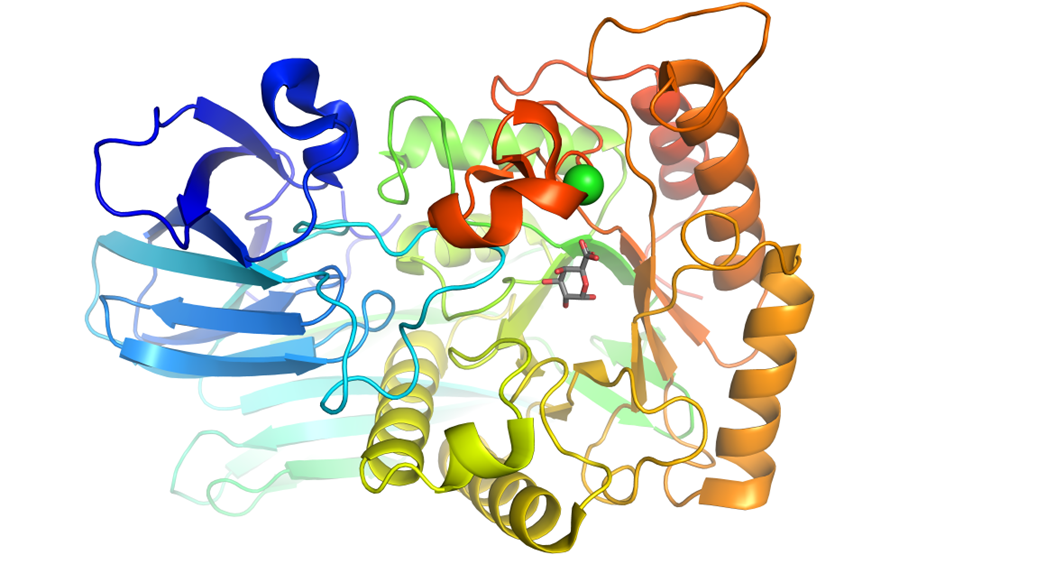


**Supplementary Figure S7. Structure of the ꞵ-galacturonidase from *E. tayi* (6NCX) belonging to the GH2_3 subfamily.** The structure was solved in the presence of Chlorure ion (green ball) and aDGlcpA (grey stick).

**
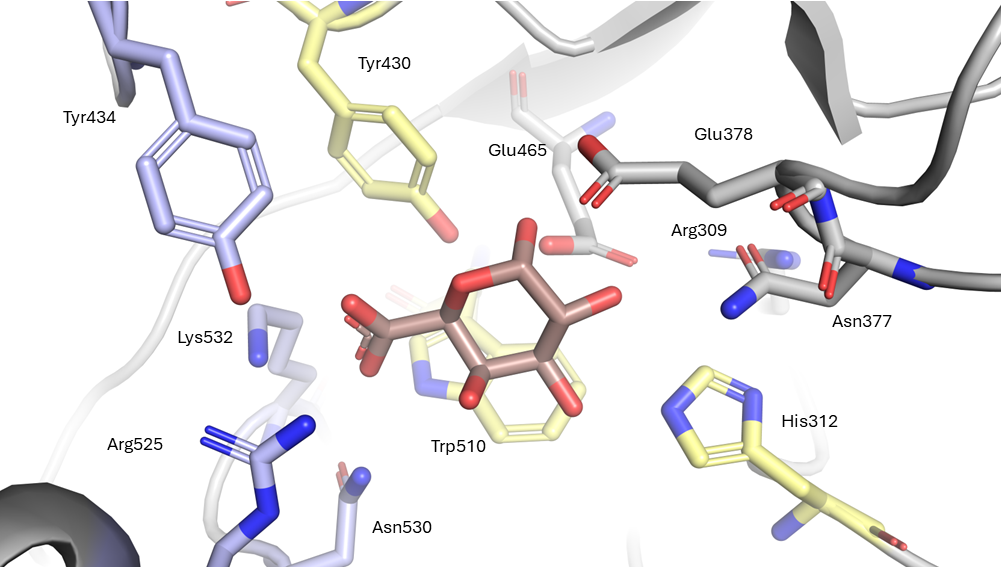
**

**Supplementary Figure S8. Active site of the GH2_3 ꞵ-galacturonidase from *E. tayi* (6NCX).** The galacturonic acid is at the center (in red). Amino acids involved in the stabilization of the carboxylate function are indicated in blue, catalytic amino acids in grey, and the conserved positions in the surrounding environment in yellow.


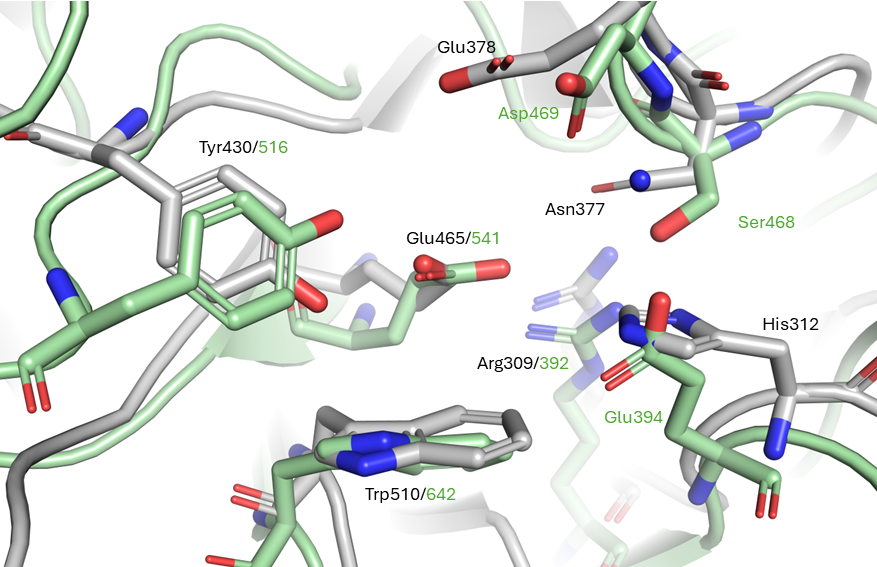


**Supplementary Figure S9. Superposition of the ꞵ-galacturonidase from *E. tayi* (6NCX) in GH2_3 (grey), with the ꞵ-glucosaminase from *A. orientalis* (2X09) in GH2_16 (green).**


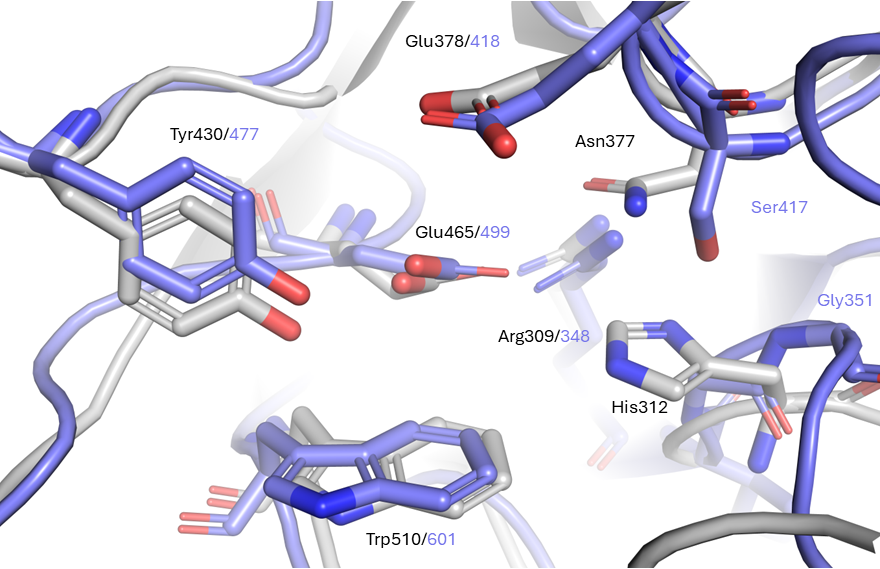


**Supplementary Figure S10. Superposition of the ꞵ-galacturonidase from *E. tayi* (6NCX) in GH2_3 (grey), with the ꞵ-galactosidase from *S. degradans* (Q21PE6) in GH2_14 (blue).**

**
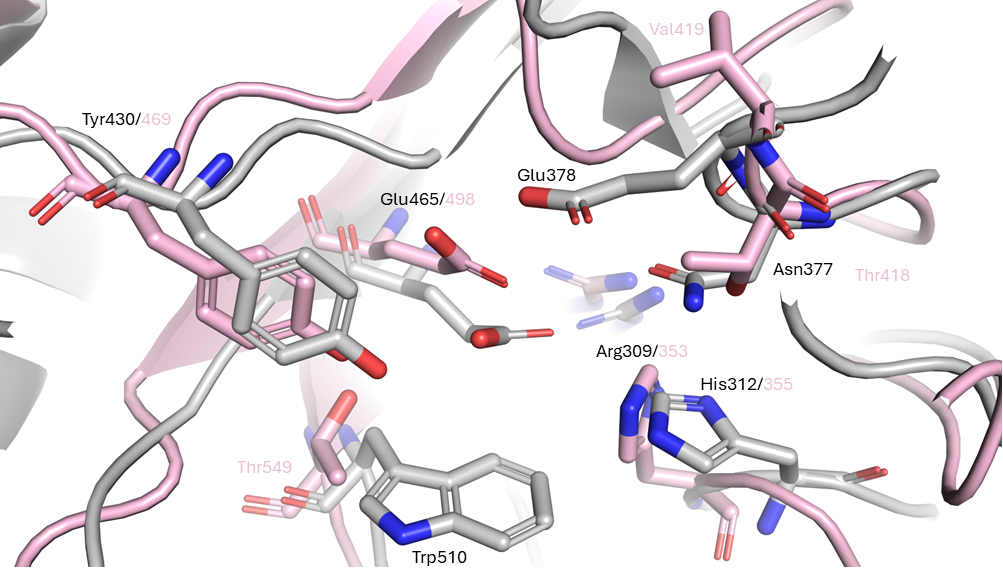
**

**Supplementary Figure S11. Superposition of the ꞵ-galacturonidase from *E. tayi* (6NCX) in GH2_3 (grey), with the uncharacterized 7XYR from *B. thetaoitaomicron* in GH2_19 (pink).**

**
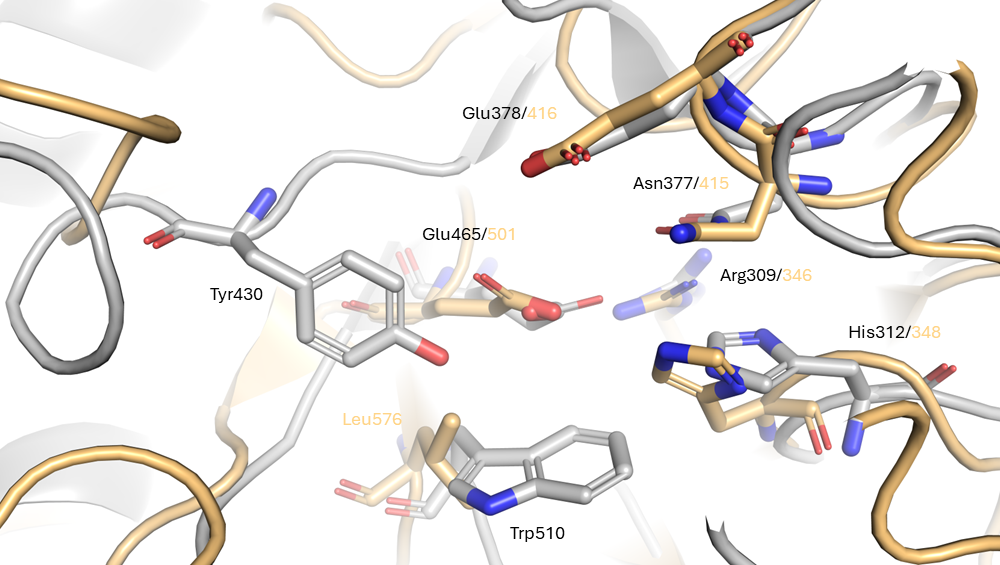
Supplementary Figure S12. Superposition of the ꞵ-galacturonidase from *E. tayi* (6NCX) in GH2_3 (grey), with the uncharacterized 8U01 from *P. plebeius* in GH2_20 (orange).**
