# Supplementary material for: Division of the large and multifunctional glycoside hydrolase family 2: high functional specificity and biochemical assays in the uncharacterized subfamilies
Source: Biotechnol Biofuels Bioprod. 2025 Jul 9;18:68. doi: 10.1186/s13068-025-02669-8 (PMC12243196; doi:10.1186/s13068-025-02669-8)
Supplement: Supplementary file 2 — Additional file 2. [file 13068_2025_2669_MOESM2_ESM.docx]

Detailed descriptions of GH2 subfamilies

GH2_1

The GH2_1 members are mostly encoded by Bacteroidota (~40%) and, to a lesser extent, by Bacillota, Gammaproteobacteria and Fungi (almost exclusively in Ascomycota). GH2_1 catalytic modules can sometimes (<10%) be associated with carbohydrate-binding modules (CBMs), mainly in Bacillota which frequently harbor one or two CBM71 and other interspersed modules. Such modular architecture frequently leads to proteins longer than 2,200 amino-acids (aa), such as the β-galactosidase BgalA from *Streptococcus pneumoniae* contributing to its virulence and which CBM71 was shown to bind lactose and LacNAc (Galβ1-4GlcNAc). Apart from the five characterized orthologs in *S. pneumoniae* strains, two additional shorter CBM71-containing GH2_1 were characterized as β-galactosidase in *Nialla circulans* strains*,* another Bacilli*.* More rarely, other CBMs were observed, such as a CBM35 in Bacteroidota or a CBM32 in Actinomycetota which include three characterized β-galactosidases in *Bifidobacterium bifidum* strains (one described as lactose-specific, EC 3.2.1.108). The majority of GH2_1 members, devoid of CBMs, are rather between 800-900 aa long, and are also reported as β-galactosidases (16 cases) with one noteworthy exception. Indeed, the Actinomycetota Bgp2 encoded by *Microbacterium esteraromaticum* was shown to display an α-L-arabinopyranosidase activity. Such activity is also reported in the sole characterized GH2_6 member. 3D structures have been solved for seven β-galactosidases GH2_1 (including proteins with CBM71, CBM32, or without CBMs) as well as for two uncharacterized ones.

GH2_2

The GH2_2 members are mostly encoded by Bacteroidota (~60%) and to a lesser extent by Bacillota (mostly Clostridia). These proteins displayed an additional C-terminal CBM57 domain sometimes (<20%), notably in most Alpha- and Betaproteobacteria members, leading to proteins of around 880 aa, *i.e.* 200 aa longer than the average. Thirteen members were functionally characterized from Bacteroidota, Clostridia and environmental samples, all displaying a β-glucuronidase activity. Nine of these also had their 3D-structure solved, as well as two uncharacterized GH2_2. Functionally and structurally characterized members include proteins with and without CBM57s.

GH2_3

The GH2_3 members are encoded by a wide range of organisms, Gammaproteobacteria (~40%) and Bacillota (~25%) for Bacteria, and Fungi (15%) and Metazoa (5%) for Eukaryota. These proteins do not contain other known catalytic domains nor CBMs, having the shortest average length among GH2 subfamilies around 530 amino-acids. Thirty-six GH2_3 members have been characterized in various organisms, mostly Bacillota (44%), Fungi (19%) and Metazoa (14%) while a single Gammaproteobacteria. All exhibit a β-glucuronidase activity except two reported β-galacturonidases. The taxonomic diversity likely accompanies substrate diversity, from the human lysosomal enzyme involved in the degradation of glucuronate-containing glycosaminoglycans, to the β-glucuronidase exploited for the conversion of glycyrrhizin, an edulcorant extracted from licorice, by Fungal GH2_3 members. The β-galacturonidase activity reported in two human gut Bacillota (Clostridia) well demonstrates the structural differentiation between β-glucuronidase and β-galacturonidase. Eighteen members of GH2_3 have a resolved 3D structure, fifteen of which among the functionally characterized members.

GH2_4

The GH2_4 members are mostly encoded by Bacteroidota (~60%) and Pseudomonadota (>30%, mainly from Alphaproteobacteria). These proteins do not contain other known catalytic domains nor CBMs, having an average size of around 600 amino-acids. Five of the GH2_4 members were characterized including four with a resolved 3D structure. All of them displayed a β-glucuronidase activity, while two of them have a supplementary β-galacturonidase activity. Of note, the characterized proteins are encoded by bacteria from different phyla and ecosystems: two gut *Bacteroides* and a marine *Aquimarina* for the Bacteroidota and species sampled from gut and compost for the Baccillota (*Clostridia*).

GH2_5

The GH2_5 members are mostly encoded by Alphaproteobacteria and Bacillota representing ~80% of the subfamily. These proteins do not contain other known catalytic or binding domains, leading to sequences between 750 and 800 amino-acid long. Five members were functionally characterized as β-galactosidases, originating from two soil Alphaproteobacteria and two Bacillota (Clostridia, genus *Thermoanaerobacter*) and a marine Gammaproteobacteria active on agar oligosaccharides. Only one 3D structure was solved for the cold-adapted protein ParβDG in soil-sampled *Paracoccus sp. 32d*.

GH2_6

The GH2_6 members are mostly encoded by Bacteriodota (>75%). These proteins sometimes display an additional C-terminal CBM32 domain (~10%), leading to proteins around 970aa, *i.e*. 100 aa longer than the average. A single protein was functionally characterized as an α-L-arabinopyranosidase during rhamnogalacturonan-II degradation by the model gut *Bacteroides thetaiotaomicron*. Such activity is only found in one GH2_1 (otherwise β-galactosidases) member which however acts on ginsenosides. No member is structurally characterized to date.

GH2_7

The GH2_7 members are mostly encoded by Bacteroidota (>75%). These proteins do not contain other known catalytic or binding domains, leading to sequences around 800 amino-acid long. This subfamily does not contain any structurally characterized member.

GH2_8

The GH2_8 subfamily is almost restricted to Bacteroidota, but a single member is found in a Verrucomicrobia. All these proteins are multimodular and display two C-terminal modules from families CBM57 and recently created CBM97. Members encoded by gut-colonizing species are fused with an additional GH137 catalytic module at the N-terminus, leading to protein length around 1410 aa. This is notably the case of the sole characterized GH2_8 member in which these two GH modules were shown to cleave moieties in two distinct chains of the RG-II, the GH2_8 β-glucuronidase acting on chain A and the GH137 β-L-arabinofuranosidase on chain B. Interestingly, in "environmental" species, GH2_8 domains are not fused to a GH137 but frequently display an independent GH137 in their PULs. Additionally, most display an additional module in-between the two CBMs, having distant homology to some modules of unknown function attached to CE and PL modules, leading to proteins of around 1170 aa. No structure was solved for any GH2_8 domain, while the GH137 and CBM97 were.

GH2_9

The GH2_9 members are mostly encoded by Bacteroidota and Verrucomicrobia (>90% in similar amounts), however largely influenced by dozens of strains from *Bacteroides fragilis* and *Akkermansia muciniphila*. Most of these proteins display a C-terminal CBM32 domain, leading to proteins around 860 aa. The GH2_9 subfamily did not contain any structurally characterized members.

GH2_10

The GH2_10 members are encoded by a wide range of organisms including bacteria, eukaryotes and rare viruses. Most bacterial members belong to the Gammaproteobacteria, followed by Actinomycetota, Bacillota and Bacteroidota, all these summing up to ~80%. Eukaryotic sequences are mostly encoded by Fungi (>10%) from Ascomycota. These proteins rarely contain other known catalytic or binding domains (<3%) but are encoded by proteins longer than 1000 aa. A β-galactosidase activity was reported for 73 of them, both in bacteria (68; ~20 for each Gammaproteobacteria, Actinomycetota and Bacillota-Bacilli, while only 2 in Bacteroidota) and fungi (5) isolated from dairy products, infant gastrointestinal tract, rumen, other feces and isothermal cold environments such as polar regions and deep-sea. Eight members of the GH2_10 have a 3D structure resolved, five among the functionally characterized while two unpublished structures are notably from the model human gut *Bacteroides thetaiotaomicron*.

GH2_11

The GH2_11 members are dominated by Bacteroidota (~90%). These proteins do not contain other known catalytic or binding domains, leading to protein length around 930 aa. No 3D structure was solved while two GH2_11 has been characterized in the model gut *Bacteroides thetaiotaomicron*: one β-galactosidase removing decorations from rhamnogalacturonan type-I and β-galacturonidase implied in the degradation of the chain C of rhamnogalacturonan type-II. Interestingly, β-galacturonidase activities reported in other GH2 subfamilies fall into GH2_3 and GH2_4 which are dominated by β-glucuronidases.

GH2_12

The GH2_12 members are mainly encoded by Bacteroidota (>80%). These proteins do not contain other known catalytic or binding domains despite a rather average protein length >1000 aa. This subfamily does not contain any structurally nor functionally characterized members to date.

GH2_13

The GH2_13 members are mostly encoded by Fungi (~60%) of the Ascomycota phylum and by a wide range of bacteria (Actinomycetota, Alphaproteobacteria, Bacillota, Bacteroidota and Gammaproteobacteria). These proteins do not contain other known catalytic or binding domains, leading to proteins around 870 amino-acid long. Among their members, 26 have a β-mannosidase activity. They are encoded by diverse bacteria (13) including thermophilic species of terrestrial and marine environment, by fungi (8) mainly from the Aspergillus and Trichoderma genera, and by Metazoa (5) including Human, cow and a marine gastropod. One of them, encoded by the thermophilic Bacilliota *Caldicellulosiruptor bescii* was also reported to bind to insoluble galactomannan in a pH-dependent fashion^6^. The 3D structure of four of these β-mannosidases were resolved.

GH2_14

The GH2_14 members are mostly encoded by Pseudomonadota, Alphaproteobacteria and Betaproteobacteria each gathering >40%, and to a lesser extent by Actinomycetota (>10%). These proteins do not contain other known catalytic or binding domains, leading to proteins around 820 amino-acid long. The taxonomic distribution in this subfamily reveals an oversampling of a few genera, notably *Agrobacterium*, *Burkholderia* and *Rhizobium*-related. This subfamily does not contain any structurally characterized members so far.

GH2_15

The GH2_15 members are mostly encoded by Gammaproteobacteria (>90%). These proteins do not contain other known catalytic or binding domains, having sequences around 720 amino-acids. Three members were characterized as β-glucosidase in three species of the *Thermus* genus (Deinococcota phylum). Of note, the *Thermus thermophilus* protein TTP0222 assays on various pNP substrates revealed additional weak but higher levels of activity as β-N-acetylhexosaminidase and β-galactosidase.

GH2_16

The GH2_16 members are mostly encoded by Fungi (>70%) from Ascomycota and, to a lesser extent, by Actinomycetota (15%). These proteins display in rare cases (<2%) an additional C-terminal CBM35 domain and are around 880 amino-acid long. This subfamily has five members functionally characterized as exo-β-1,4-glucosaminidase (3.2.1.165), three from Fungi and two from Actinomycetota. One of the characterized GH2_16 in Actinomycetota displays a C-terminal CBM35. This protein is also the sole structurally characterized GH2_16, and has been shown to hydrolyze chitosan present in Fungi.

GH2_17

The GH2_17 members are mostly encoded by Bacteroidota and, to a lesser extent, by Gammaproteobacteria, both representing ~80% of the members, and notably in some Viridiplantae. These proteins are frequently combined (>30%) with CBM32 modules but it seems mainly restricted to Bacteroidota (85%). These CBM32 can be found either at the N- or C-terminus of the proteins, sometimes with a tandem duplication, forming sequences in average >1000 amino-acid long. This subfamily does not contain any structurally characterized member but three functionally characterized GH2_17 show a mannosyl-glycoprotein endo-β-mannosidase activity. To be noted, all three characterized members are all encoded by Viridiplantae genomes from the Mesangiospermae and none bear a CBM32 module.

GH2_18

The GH2_18 members are mostly (~90%) encoded by Actinomycetota, Bacteroidota, and Fungi (mainly from Ascomycota) in comparable amounts. These proteins display an average length around 680 aa and are, in rare cases (~6%), combined with CBMs. The most prevalent case (93% of multimodular ones, mostly from Actinomycetota) is a combination with one to two copies of a CBM42, a module reported binding to arabinofuranose. The five biochemically characterized GH2_18 have a β-D-galactofuranosidase activity. Three originate from Fungi, including one bi-functional α-L-arabinofuranosidase, and two from Actinomycetota including one fused to a CBM42. A single GH2_18 has been structurally characterized, but not among the functionally characterized members.

GH2_19

The GH2_19 members are mostly encoded by Fungi (~40%, mainly Ascomycota) and Bacteria from Actinomycetota, Bacillota and Bacteroidota phyla (~50%). These proteins do not contain other known catalytic or binding domains and form proteins of ~650 amino-acids. Four GH2_19 were functionally characterized with three different activities in various phyla: a fungal β-1,5-D-galactofuranosidase, an hyperthermophilic (Thermotogae) α-L-arabinofuranosidase activity – both activities shared only with GH2_18 – and two β-galactosidase activities reported in Actinomycetota and Alphaproteobacteria phyla. The sole 3D structure of a GH2_19 member was resolved in the model human gut *Bacteroides thetaiotaomicron* but without functional characterization to date, and a likely erroneous automatic annotation in non-specialist databases (β-glucuronidase).

GH2_20

The GH2_20 members are mostly encoded by Bacteroidota (>80%) and to a lesser extent by Bacillota (~10%). These proteins, rarely combined with other known modules but in 2% with a CBM32, display an average size ~930 aa. This subfamily has not been structurally characterized but two members were functionally characterized with a β-galactosidase activity in the model human gut *B. thetaiotaomicron* and a compost-isolated Bacillota, while another member showed an undetermined activity on xyloglucan. This ambiguity could be explained if the enzyme is also a β-galactosidase catalyzing the hydrolysis of terminal β-(1,2)-linked galactosyl residues from tamarind xyloglucan.

GH2_21

The GH2_21 members are mostly encoded by Bacteroidota (73%) and to a lesser extent by Fungi (16%, mostly due to *Aspergillus* genus). These proteins do not exhibit any additional catalytic nor binding domains, sequences being around 960 amino-acid long. This subfamily has four members functionally characterized as β-glucuronidases, all from Bacteroidota from distinct environments. For example, in the model gut *B. thetaiotaomicron,* the GH2_21 has been shown to cleave branched arabinan, while in the marine *Formosa agariphila* uses a GH2_21 during ulvan breakdown and the natural substrate of the soil *Niabella aurantiaca* remains to be determined. Only the *F. agariphila* ulvan-processing GH2_21 has been structurally characterized.

GH2_22

The GH2_22 members are mostly encoded by Bacteroidota (>80%, mostly from *Bacteroides* and *Phocaeicola* species/strains). These proteins do not contain other known catalytic or binding domains despite a rather long sequence around 1050 amino-acids. This subfamily contains a single characterized member in human gut *Bacteroides cellulosilyticus* that displays a β-xylosidase activity, unique in the GH2 family. No structurally characterized member was reported.

GH2_23

The GH2_23 members are mostly encoded by Bacteroidota (95%). These proteins do not exhibit any other known catalytic nor binding domains while reaching an average length of 1064 amino-acids. The GH2_23 subfamily does not contain any structurally characterized members.
